# Work-focused cognitive behavioural therapy for common mental disorders: a systematic review and meta-analysis

**DOI:** 10.1186/s40359-026-05648-2

**Published:** 2026-09-22

**Authors:** Anna Finnes, Magnus Johansson, Robin Bernhardtz, Lars-Göran Öst

**Affiliations:** 1 Department of Clinical Neuroscience, Division of Insurance Medicine, Karolinska Institutet, Stockholm, Sweden; 2 Academic Primary Health Care Center, Region Stockholm, Stockholm, Sweden; 3 Department of Clinical Neuroscience, Centre for Psychiatry Research, Karolinska Institutet, Stockholm, Sweden; 4 Department of Psychology, Stockholm University, Stockholm, Sweden

**Keywords:** Return to work, Sick leave, Mental disorders, Psychological treatment, Bounded data, Zero-inflation, Occupational health

## Abstract

**Background:**

Common mental disorders (CMDs) are a major cause of sickness absence and work disability. Although cognitive behavioural therapy (CBT) effectively reduces symptoms, effects on return-to-work (RTW) outcomes are limited. Work-focused CBT (W-CBT) integrates RTW processes into treatment, but the evidence base remains heterogeneous with unresolved methodological challenges. This systematic review and meta-analysis evaluated the effectiveness of W-CBT on psychiatric and work-related outcomes, examined moderators of treatment effects, assessed methodological rigour, and identified common intervention components, with particular attention to how RTW outcomes are analyzed.

**Methods:**

Systematic searches were conducted in Medline, PsycINFO, and Web of Science from inception to December 2025. Randomized and non-randomized studies evaluating CBT with an integrated work focus among working-age adults on sickness absence due to CMDs were included. Random-effects meta-analyses were performed using multilevel models with cluster-robust standard errors to address outcome dependency. Moderator analyses examined treatment content, number of sessions, and study characteristics. RTW outcomes were evaluated regarding their statistical properties, summary measures, and analytic methods used in primary studies. Risk-of-bias was assessed using Cochrane Risk of Bias tool version 2 and ROBINS-I.

**Results:**

Twenty-four studies comprising 26 W-CBT treatment conditions (*n* = 3,279 participants) were included. Large within-group improvements in psychiatric symptoms were observed and maintained at follow-up, while between-group effects were statistically significant but small. At post-treatment, 56% of participants had returned to work, increasing to 87% at follow-up, with a higher likelihood of full RTW in W-CBT compared with controls. CBT content, stronger work focus, and higher treatment dose were associated with larger symptom improvements. However, RTW outcomes showed substantial heterogeneity. Many studies used analytic approaches assuming normally distributed data for outcomes that were bounded, skewed, or zero-inflated, or reported hazard ratios with limited comparability, constraining valid synthesis.

**Conclusions:**

W-CBT is associated with meaningful symptom improvement and increased likelihood of RTW. However, misalignment between RTW outcome properties and analytic methods represents a major threat to validity. Future research requires improved outcome definitions, appropriate statistical modeling, and greater methodological consistency.

**Supplementary Information:**

The online version contains supplementary material available at https://doi.org/10.1186/s40359-026-05648-2.

## Introduction

Common mental disorders (CMDs), particularly depressive, anxiety, and stress-related disorders, are highly prevalent in the working-age population and constitute a major contributor to global disability and societal burden [1, 2]. Large cross-national surveys indicate that approximately 30% of adults meet criteria for a mental disorder during their lifetime, while cumulative risk by age 75 approaches 50% [1]. Gender differences include higher lifetime risk, longer sickness absence (SA) durations, and lower early recovery rates among women, among whom mood and anxiety disorders are also more common [1, 3]. In Europe, CMDs affect approximately one in six adults annually and account for the majority of the overall mental health burden [4].

CMDs are associated with substantial impairments in functioning and quality of life [5], long-term unemployment, sickness absence, and disability pension, suggesting a potential path to labour market marginalization [6]. Evidence from large outpatient psychiatric samples shows that more than 70% of patients report severe quality-of-life impairment and approximately 60% report severe functional impairment at treatment entry, particularly among individuals with mood disorders [7]. Functional impairments commonly affect cognitive, emotional, social, and occupational domains, making CMDs a leading cause of sickness absence and work disability in high-income countries [8]. Sickness absence due to CMDs is often prolonged and recurrent, with delayed or incomplete return to work (RTW) compared with many somatic conditions [9]. Psychosocial work stressors, including effort-reward imbalance, job strain, low job control, and high psychological demands, further exacerbate symptoms and increase the risk of sickness absence among individuals with CMDs [10], illustrating the bidirectional relationship between CMDs and work disability.

Cognitive behavioural therapy (CBT) is an established, evidence-based first-line treatment for depression and anxiety disorders [11]. Whereas treatment can effectively reduce the acute symptoms of a disorder (e.g., mood, anxiety, fatigue), individuals may still face challenges in returning to their previous level of performance in complex, real-world settings such as the workplace [12]. Further, job performance may remain impaired even after symptoms have improved [13]. Work-focused CBT (W-CBT) has been developed by integrating return-to-work processes directly into CBT treatment [14, 15]. In W-CBT, work participation is framed as a central therapeutic goal and used as an active context for applying CBT strategies, including restructuring work-related beliefs, behavioural experiments, graded exposure to work demands, and structured problem-solving related to workplace barriers [15].

Previous systematic reviews suggest that W-CBT interventions for mental disorders may improve return-to-work outcomes [16], whereas others point to substantial heterogeneity across interventions, study populations, and RTW outcome measures, precluding meta-analysis [15, 17]. Another likely explanation for the mixed findings is heterogeneity across studies with respect to intervention components, study design, therapist competence, and outcome definitions. For example, psychological interventions appear to reduce sickness absence in depression but not in adjustment disorders [18, 19]. Previous reviews have also highlighted variability in methodological quality, limiting firm conclusions regarding effectiveness and key intervention components.

To advance the field, a systematic review and meta-analysis is warranted that explicitly examines W-CBT in relation to both clinical and work-related outcomes, explores potential moderators of treatment effects, and systematically maps methodological limitations as well as common core components of W-CBT interventions.

The objectives of this systematic review and meta-analysis are:

- To evaluate the current state of evidence of W-CBT in improving work functioning and psychiatric symptoms for common mental disorders.
- To investigate potential moderators of treatment outcome.
- To provide an evaluation of methodological stringency in the studies.
- To investigate the common core components of W-CBT.

## Methods

The protocol for this meta-analysis was pre-registered at PROSPERO with ID CRD42021265973. The review follows the Preferred Reporting Items for Systematic Reviews and Meta-Analyses (PRISMA) 2020 guidelines [20]. The study was funded by Afa Insurance (Grant number 200051). The meta-analysis was designed according to the PICOS acronym in the following way.

- *Population*: working age adults (18 to 65 years) on SA due to CMDs (i.e., mild to moderate symptoms of depression and anxiety disorders or conditions related to stress such as adjustment disorder or burnout). Employment status was not restricted; participants could be employed, self-employed, unemployed, or receiving sickness benefits. Studies focusing on severe mental disorders (e.g., psychotic disorders, bipolar disorder) or substance use disorders were excluded.
- *Intervention*: All types of cognitive behavioural therapy with an integrated work focus, defined as explicit and continuous incorporation of work participation, RTW processes, or work-related goals throughout treatment, were included.
- *Comparison*: All control conditions were accepted, including psychological or non-psychological treatments, treatment as usual, and waitlist. If a psychological treatment was compared to another psychological treatment within the same study, the experimental treatment and control group as chosen by the authors of that study were considered active treatment and comparison group, respectively.
- *Outcome*: Primary outcomes were changes in psychiatric symptom measures and work-related measures. Symptom measures could be self-report scales or interview-based rating scales. Work-related measures included return-to-work, time to partial or full RTW, cumulative duration of sickness absence, recurrence of sickness absence, or changes in working hours.
- *Study design*: randomized controlled trials (RCTs), non-randomized controlled trials, and pre-post trials were included.

### Literature search

Eligible studies were identified through searches of Medline (Ovid), PsycINFO (Ovid for the original search and ProQuest for updates), and Web of Science. Key articles informed the selection of MeSH and free-text terms, which were pilot-tested in Medline and adapted to the controlled vocabularies of PsycINFO; corresponding free-text terms were used in Web of Science. Wildcards and adjacency operators were applied as appropriate. The search strategy was developed iteratively in collaboration with librarians from Karolinska Institutet University Library. The initial search was conducted in March 2021 and the final update was performed on 8 December 2025, following established methods for updating systematic searches [21]. In total, 2,732 database records were assessed.

The search was based on two main themes: cognitive behavioural therapy and return-to-work/work focused treatment. For the full search strategy for all text words/subject headings searched in all three databases, see Additional file 1. We also went through references of included articles and their citations in Web of Science.

Two authors (AF, RB) independently screened all abstracts to determine eligibility for full-text review. Screening was intentionally over-inclusive; any indication of a work-focused cognitive behavioural intervention prompted full-text retrieval. Reference lists of retrieved articles were hand-searched to identify additional eligible studies. Final inclusion was based on predefined inclusion and exclusion criteria. Full texts were reviewed by AF and RB, with disagreements resolved by consensus and, when necessary, consultation with the last author. Ultimately, 24 articles comprising 26 treatment conditions were included in the meta-analysis.

#### Inclusion criteria

Studies were eligible for inclusion if they met the following criteria: (1) published or in press in an English-language journal; (2) included working-age adults (18–65 years) on sickness absence due to common mental disorders (i.e., mild to moderate depression or anxiety, or stress-related conditions such as adjustment disorder or burnout); (3) evaluated a CBT-, CT-, or BT-based intervention with an integrated work focus; (4) included at least 10 treated participants; and (5) reported at least one continuous or dichotomous outcome of mental health or work status, including any measure of RTW or sickness absence (e.g., time to full or partial RTW, days of sickness absence, or hours worked).

**Fig. 1:**
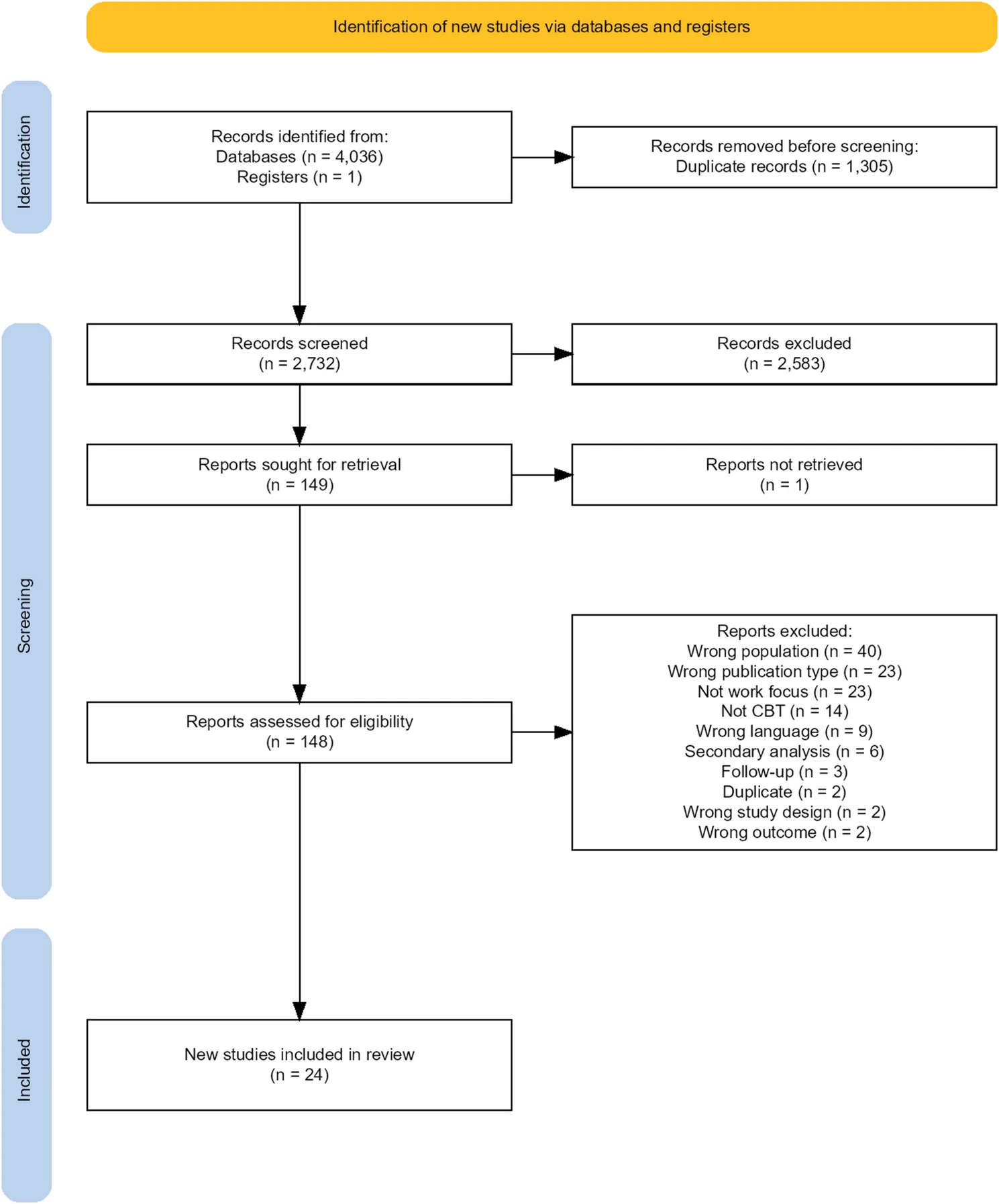
PRISMA flowchart

Secondary analyses of previously published studies were excluded, whereas follow-up publications of eligible primary studies were included when they reported additional long-term outcome data. Figure 1 presents the study selection flowchart. References for included studies are provided in Additional file 2.

### Data extraction

The first and third authors independently extracted data using a standardized form covering study characteristics, methods and interventions, moderator variables, and outcome data. Discrepancies were resolved by consensus discussion or, when necessary, consultation with the last author.

### Potential categorical moderators

Potential categorical moderators were included only if at least 70% of the studies reported the variable, to ensure representativeness. In addition, the smallest category within a moderator was required to include at least three studies.

#### Mental disorder

Type of mental disorder was categorized as adjustment disorder, stress, depression, social anxiety disorder, or mixed disorders (anxiety, depression, and adjustment disorders), as defined by the original study authors.

#### Study design and statistical analysis

Study design was classified as randomized controlled trial (RCT; CBT compared with a control or comparison condition) or non-RCT (non-randomized controlled or pre–post designs with a CBT condition only). Statistical analysis was categorized as completer analysis (dropouts excluded) or intention-to-treat (ITT; all randomized or enrolled participants included).

#### Treatment format and therapist profession

Treatment format was categorized as individual or group. Therapist profession was classified by the professional background of the therapists; studies involving multiple professions were categorized as mixed.

#### Setting and workplace intervention

Study setting was categorized as clinic, occupational health service, or university. Workplace intervention was coded as yes when therapists had contact with the participant’s workplace and no when no such contact occurred.

### Potential continuous moderators

Continuous moderators were included if at least 70% of studies reported the variable. These comprised publication year, sample size, percentage of female participants, mean age, number of treatment sessions, percentage on sickness absence, methodology score, and treatment content scores (see below). Attrition rates were also extracted for each treatment condition. Dropouts were defined as participants who met the inclusion criteria, accepted treatment, attended at least one session, but did not complete the number of sessions specified as treatment completion by the study authors. Data extraction and coding were performed independently by authors AF and RB, with disagreements resolved by consensus.

### Methodological quality

#### The psychotherapy outcome study methodology rating scale (POMRS)

The scale comprises 22 items assessing key methodological aspects of psychotherapy outcome research [22]. Each item is rated on a 3-point scale (0 = poor, 1 = fair, 2 = good) with explicit verbal anchors. Item descriptions and study ratings are presented in Additional file 3, Fig. 3. Inter-rater reliability between the first and last author, based on 20% of the studies, randomly selected and rated independently and blinded, was excellent (ICC = 0.94, 95% CI [0.48, 0.99], *p* = 0.007), which is considered excellent according to Cicchetti [23].

#### Treatment content assessment

Treatment content was assessed using a study-specific rating scale developed for this meta-analysis. The scale comprised 19 items: 11 items assessing standard CBT procedures and seven items assessing workplace implementation (forming the two subscales), plus one item capturing the integration of CBT and workplace components (see Additional file 3, Figs. 1 and 2 for details). Items were rated Yes (1 point) or No (0 points) and summed separately for each subscale. All studies were independently rated by the first and second authors prior to outcome data extraction, ensuring blinded assessment. A coding scheme and scoring manual specifying all variables were developed to guide ratings.

#### Risk-of-bias

Risk of bias was assessed in accordance with study design using established tools. Randomized controlled trials were evaluated using a subset of domains from the Cochrane Risk of Bias tool, version 2 [24], assessing bias related to the randomization process, missing outcome data, outcome measurement, and selective reporting. The domain concerning deviations from intended interventions was not evaluated, as blinding of therapists and participants is not possible in psychotherapy trials. Non-randomized studies and pre–post designs were assessed using the ROBINS-I tool [25], covering bias due to confounding, participant selection, intervention classification, deviations from intended interventions, missing data, outcome measurement, and selective reporting.

For randomized trials, overall risk of bias was categorized as either low risk, some concerns, or high risk. For non-randomized and pre–post studies, judgments were classified as low, moderate, serious, or critical risk of bias. To support synthesis across study designs, these ratings were subsequently harmonized into three overarching categories: low, moderate (including “some concerns”), and high risk of bias (including “serious” and “critical” ratings).

### Effect size measures

#### Measures of psychiatric symptoms

Anxiety outcomes were reported in 14 studies: five used the Beck Anxiety Inventory [26], three the Hospital Anxiety and Depression Scale–Anxiety [27], two the Four-Dimensional Symptom Questionnaire–Anxiety [28], two the Depression Anxiety Stress Scale–Anxiety [29], one the Symptom Checklist-90–Anxiety [30], and one the Generalized Anxiety Disorder-7 [31].

Depression was assessed in 17 studies, most commonly with the Beck Depression Inventory-II [32] (*n* = 6), followed by the Patient Health Questionnaire-9 [33] (*n* = 3). The Hospital Anxiety and Depression Scale–Depression [27] was used in two studies, the Four-Dimensional Symptom Questionnaire–Depression [28] in two, and the Center for Epidemiologic Studies Depression Scale [34], the Depression Anxiety Stress Scale–Depression [29], the Montgomery Åsberg Depression Rating Scale–Self-rated [35], and the Symptom Checklist-90–Depression [30] were used in one study each.

Stress was measured in six studies: five used the Perceived Stress Scale [36], and one the Depression Anxiety Stress Scale–Stress [29]. Exhaustion was assessed in four studies: three used the Maslach Burnout Inventory [37] and one the Karolinska Exhaustion Disorder Scale [38]. General mental health was measured in two studies using the Symptom Checklist-90 [30]. Distress and social anxiety were each assessed in one study, using the Kessler-6 Distress Scale [39] and the Liebowitz Social Anxiety Scale [40], respectively.

#### Work measures

We extracted data for three types of measures. *SA days* were defined either as time (in days) to full RTW (reported in seven studies) or as *days on sickness absence* within a given time frame (five studies). *Proportion of RTW* was reported in 12 studies. *Work functioning*, operationalized by self-report questionnaires, was reported in five studies: three used the Work Ability Index [41], and the World Health Organization Health and Work Performance Questionnaire [42] and the Endicott Work Productivity Scale [43] were used in one study each.

### Meta-analysis

All analyses were conducted in R version 4.5.2 [44], using the packages `meta` [45], `metafor` [46], and `RoBMA` [47]. The online resource “Doing meta-analysis in R” [48] was used as a methodological reference. A flow diagram was generated using the PRISMA Flow Diagram tool [49].

For within-group continuous outcomes, standardized mean differences (SMDs) were calculated using the standard deviation of the pre-measurement as denominator, sometimes referred to as Glass’s Δ_pre_ [50, 51]. Between-group SMDs were calculated under the assumption of homoscedasticity, using the formula *SD* = $\:\sqrt{(\left(n1-1\right)*{SD1}^{2}+\:\left(n2-1\right)*{SD2}^{2})\:/\:n1+n2-2}$, where *n*1 and *n*2 denote group sample sizes for group 1 and 2, and *SD*1 and *SD*2 denote group standard deviations. Hedges’ *g* correction was applied to both within- and between-group effect sizes. A reduction in symptoms is indicated by a positive *g* value. For dichotomous outcomes, risk ratios (RR) were calculated and log-transformed prior to meta-analysis. Forest plots display log RRs, whereas RRs are reported in the text to simplify interpretation.

Intention-to-treat data were used when available; otherwise, completer data were analyzed. Random-effects models were used since it cannot be assumed that the ESs come from the same population. The inverse variance method was used to pool effect sizes, and Knapp-Hartung adjustments were used for confidence intervals.

Heterogeneity among ESs was assessed with the *Q*-, *I*^*2*^-, and τ^2^-statistics and the reporting of prediction intervals [52, 53]. Restricted Maximum Likelihood Estimator (REML) was used to estimate τ^2^ with the profile likelihood method for confidence intervals.

In meta-analyses including fewer than 10 studies, tests of publication bias and moderator effects were not performed, as these are considered unreliable [54]. Symptom outcomes included multiple measures per study (range 1–6; mean = 2.33; median = 2), necessitating 3-level random-effects models to account for non-independence while retaining correct weighting among studies [55, 56]. Cluster-robust (CR2) standard errors and confidence intervals with Satterthwaite degrees of freedom were estimated using the `clubSandwich` R package [57, 58]. Moderator analyses were conducted using mixed-model meta-regression.

The possibility of publication bias was analyzed with tests for asymmetry of funnel plots, using the Pustejovsky-Rodgers corrected standard error formula for Egger’s test [59, 60]. For proportional outcomes, Peters’ test [61] was used to evaluate asymmetry in funnel plots. It should be noted that high levels of heterogeneity combined with small number of studies reduce the power of funnel plot asymmetry tests in general [62]. For 3-level models, publication bias tests and sensitivity analyses were conducted using Robust Bayesian meta-analysis (RoBMA) [63] with a Z-curve plot [64]. RoBMA averages results from multiple meta-analytic models, each one using different assumptions regarding publication bias, heterogeneity, and effect [65, 66]. Additionally, an extension of Egger’s test for multilevel meta-analysis [67] was used to evaluate small-study publication bias.

## Results

### Description of the studies

#### Background data

Background characteristics of the included studies are presented in Table 1. In total, 24 studies were included, comprising 26 W-CBT treatment conditions, as two studies had two W-CBT interventions. The W-CBT conditions included 3,279 participants. Seven studies (29.2%) were conducted in the Netherlands, five in Denmark, four in Norway, two each in Germany, Sweden, and the United States, and one each in Canada and Japan. Diagnoses were mixed mental disorders in 13 studies (54.2%), depression in six, adjustment disorder in three, stress-related disorders in two, and social anxiety disorder in one study. Seventeen studies (70.8%) were RCTs, while seven were non-RCTs. Intention-to-treat analyses were applied in 17 studies and completer analyses in seven. Across studies, samples were predominantly female (median 70%, IQR 45–77%), with a mean age of 42.9 years (SD 3.3, range 38.5–54.7). At treatment initiation, all participants were on sickness absence in 19 of the 24 studies (79.2%).

**Table 1:** Background data of the included studies

Study	Country	Mental Disorder	# of parti- cipants	Percent women	Mean age	Design	Percent on SA	POMRS	Statistical analysis
Blonk, 2006a	Netherlands	AD	40	19.0	42.0	RCT	100	19	Completers
Blonk, 2006b	Netherlands	AD	40	19.0	42.0	RCT	100	19	Completers
Bond, 2025	Denmark	Stress	331	80.0	44.9	Non-RCT	81	13	ITT
Brouwers, 2006	Netherlands	Mixed	98	59.0	39.7	RCT	100	17	ITT
Corbière, 2021	Canada	Depression	19	90.0	43.1	Non-RCT		12	ITT
Dalgaard, 2017a; b	Denmark	Mixed	58	74.0	45.0	RCT	100	11	ITT
Dalgaard, 2014	Denmark	Mixed	57	83.9	45.0	RCT	100	10	ITT
deWeerd, 2016	Netherlands	Mixed	31	47.0	40.0	RCT	100	18	Completers
Finnes, 2017	Sweden	Mixed	88	78.4	46.3	RCT	98	26	ITT
Geraedts, 2014	Netherlands	Depression	116	62.3	43.4	RCT	0	15	ITT
Gjengedal, 2020	Norway	Mixed	87	74.9	38.7	Non-RCT	100	24	Completers
Gjengedal, 2025	Norway	Mixed	121	74.6	38.5	RCT	100	11	ITT
Himle, 2014	USA	SAD	29	32.8	43.6	RCT	0	21	ITT
Hoff, 2021	Denmark	Mixed	213	73.6	41.9	RCT	100	18	ITT
Ito, 2019	Japan	Depression	23	39.0	41.0	Non-RCT	100	8	Completers
Kröger, 2015	Germany	Depression	13	31.0	41.9	RCT	100	25	ITT
Lagerveld, 2012	Netherlands	Mixed	89	60.0	40.7	Non-RCT	100	21	ITT
Lerner, 2015	USA	Depression	217	72.0	54.7	RCT		14	ITT
Monsen, 2025	Norway	Mixed	14	43.0	43.0	Non-RCT	100	12	Completers
Noordik, 2013	Netherlands	Mixed	75	70.0	45.4	RCT	100	16	ITT
Reme, 2015	Norway	Mixed	630	67.0	40.4	RCT	100	15	ITT
Salomonsson, 2017a	Sweden	Mixed	67	82.0	42.0	RCT	100	23	ITT
Salomonsson, 2017b	Sweden	Mixed	80	82.0	42.0	RCT	100	23	ITT
van der Klink, 2003	Netherlands	AD	109	37.0	40.3	RCT	100	18	ITT
Willert, 2009	Denmark	Stress	51	82.4	45.0	RCT	67	11	ITT
Winter, 2020	Germany	Depression	20	75.0	45.8	Non-RCT	100	12	Completers

#### Treatment data

Treatment characteristics are summarized in Table 2. Interventions were delivered individually in 18 studies (75%), in groups in five, and via the internet in one. Study settings were universities in 10 studies (41.7%), clinics in eight, and occupational health services in six. Therapists were primarily psychologists in 18 studies (75%), with other professions represented in six. The median number of reported mean therapy sessions was eight (IQR 6–11; range 3.9–26). Follow-up assessments were reported for 18 treatment conditions (69.2%), with a median follow-up duration of 8.5 months (IQR = 6–12, range 3–36) after treatment completion.

**Table 2:** Treatment data of the included studies

Study	Format	Setting	Workplace Intervent.	Therapist profession	# of sessions	Time (hours)	Percent attrition	Subscale CBT	Subscale WF	Integrated	Follow-up months
Blonk, 2006a (CI)	I	OHS	Y	Other	6.0	6.0	17.5	5	1	1	6
Blonk, 2006b (CBT)	I	OHS	N	Psychologist	11.0	8.3	20.0	3	3	2	6
Bond, 2025	G	Clinic	N	Psychologist	9.0		6.3	8	1	2	36
Brouwers, 2006	I	Clinic	N	Other	4.5	4.2	0	2	2	1	15
Corbière, 2021	G	University	N	Other	6.5	6.5	6.9	7	1	2	6
Dalgaard, 2017a; b	I	University	Y	Psychologist	6.0		3.5	6	6	1	6
Dalgaard, 2014	I	University	Y	Psychologist	6.0	6.0	17.5	10	5	2	6
deWeerd, 2016	I	OHS	Y	Psychologist	19.0	19.0	12.7	2	2	0	
Finnes, 2017	I	University	Y	Psychologist	9.0	9.5	1.7	9	3	0	9
Geraedts, 2014	I	University	N	Psychologist	6.0	6.0	5.7	5	0	1	12
Gjenggedal, 2020	I	Clinic	N	Psychologist	10.4	10.4	10.3	8	5	2	
Gjengedal, 2025	I	Clinic	N	Psychologist	10	10	15.0	7	5	2	12
Himle, 2014	G	University	N	Other	7.4	16.0	4.5	4	0	1	3
Hoff, 2021	I	University	Y	Other	NI	NI	0	5	5	1	6
Ito, 2019	G	Clinic	N	Psychologist	8.0	20.0	38.2	7	0	1	
Kröger, 2015	I	OHS	N	Psychologist	22.0	22.0	10.1	10	6	2	12
Lagerveld, 2012	I	Clinic	N	Psychologist	11.1	11.1	16.4	10	6	2	6
Lerner, 2015	I	OHS	N	Other	8.0	6.7	5.0	7	3	1	
Monsen, 2025	I	Clinic	N	Psychologist	7	8.5	4.5	9	3	2	
Noordik, 2013	I	OHS	Y	Other	3.9	3.9	5.0	1	4	1	9
Reme, 2015	I	University	N	Psychologist	15.0	15.0	22.9	7	2	2	8
Salomonsson, 2017a	I	Clinic	N	Psychologist	9.1	9.1	9.8	8	4	0	12
Salomonsson, 2017b	I	Clinic	N	Psychologist	16.1	16.1	5.2	6	4	2	12
van der Klink, 2003	I	OHS	Y	Other	5.0	7.5	4.1	4	1	1	9
Willert, 2009	G	University	N	Psychologist	8.0	24.0	21.4	5	0	1	8
Winter, 2020	I	University	N	Psychologist	26.0	26.0	0	7	6	2	

#### Treatment content

The W-CBT treatment content scores for subscales were CBT mean = 6.2 (*SD* 2.5, range 1–10) and WF mean = 4.3 (*SD* 2.4, range 1–8). In 33% of the studies (*n* = 8), a workplace intervention involving a workplace representative was included. See Additional file 3, Figs. 1 and 2 for content subscale items and studies, both sorted based on total scores.

#### Work outcome data

##### Time to RTW

**Fig. 2:**
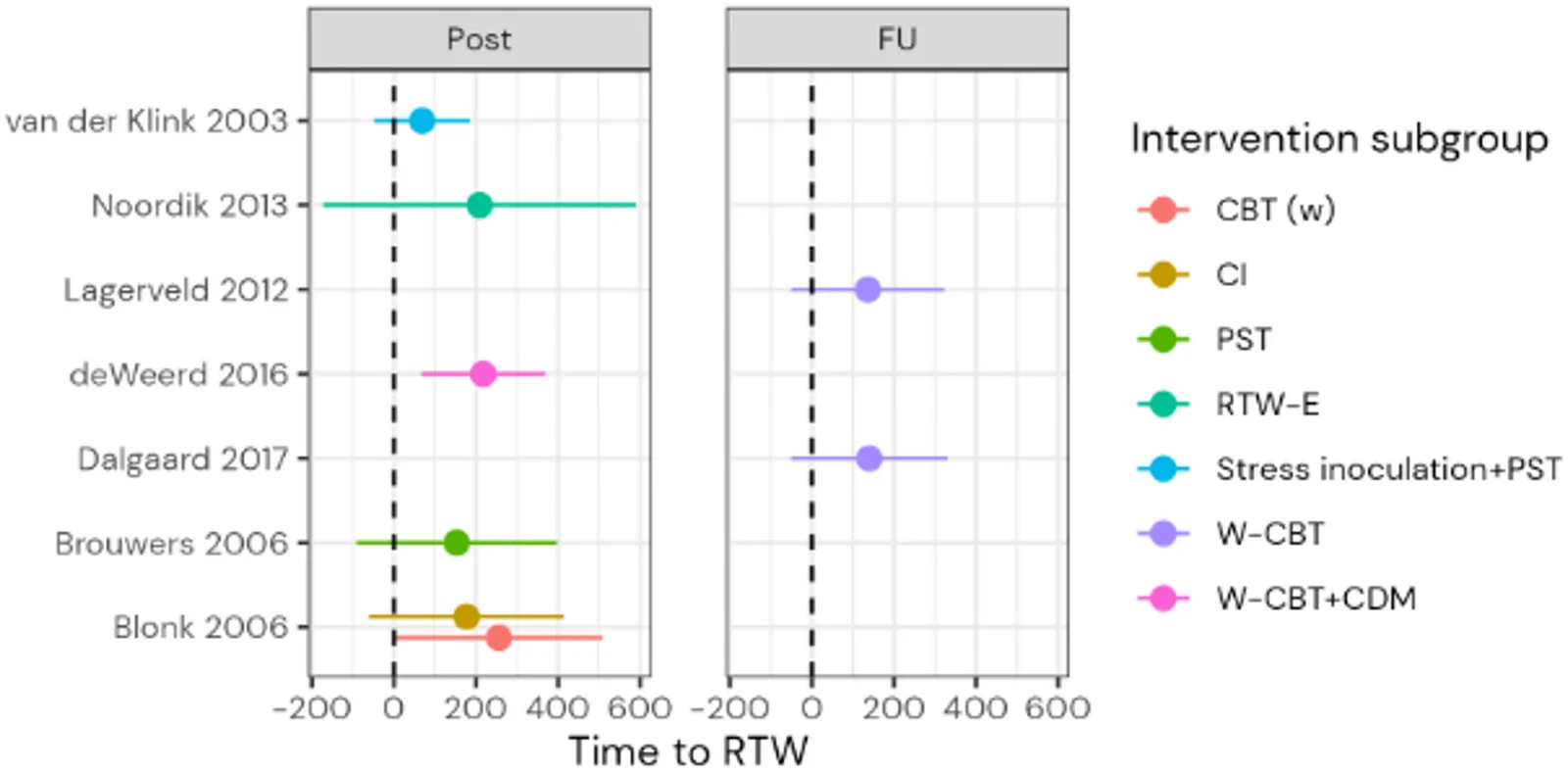
Means and 2*SD values for data on time (in days) to full RTW as reported in primary studies

SA days, operationalized as time to full RTW, were most often reported as means (or medians) with standard deviations. Because this outcome is bounded at zero and typically non-normally distributed, analysis using standardized mean differences is inappropriate. As illustrated in Fig. 2, distributions of time to RTW (in days) derived from reported means (or medians) and ± 2 SD frequently extend below zero, indicating zero inflation and likely overdispersion. Several studies analyzed time-to-event using Cox proportional hazards regression and reported a hazard ratio (HR), which was included in this meta-analysis. It should be noted that the HR is problematic in meta-analyses, not least because it is a non-collapsible measure that is dependent on the timeframe and covariates included in the analysis [68, 69]. These issues are discussed further in the Discussion section.

**Fig. 3:**
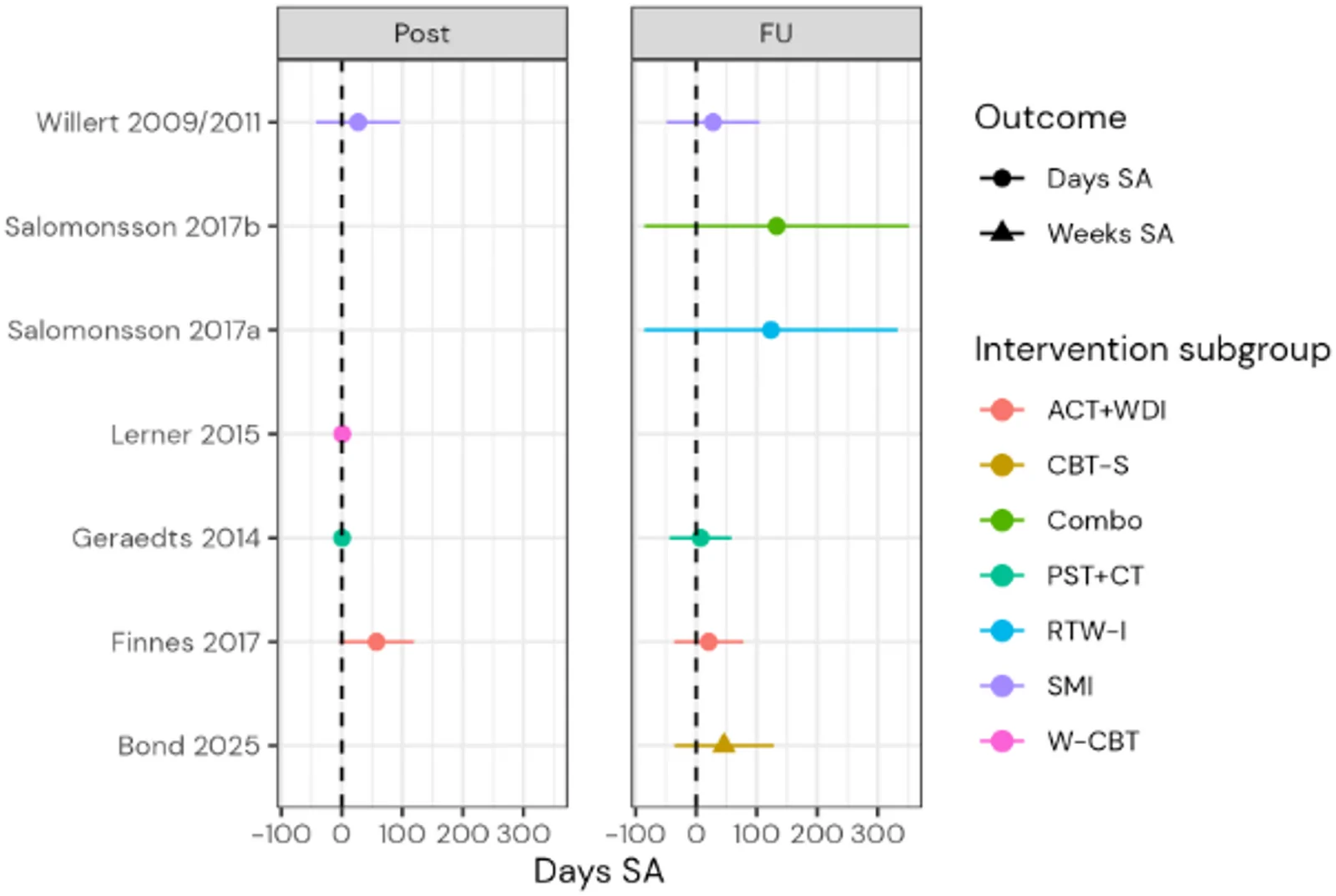
Means and 2*SD values for data on sickness absence days or weeks as reported in primary studies (x axis is cropped)

As noted above, sickness absence (SA) days are bounded at zero. As shown in Fig. 3, the lower bounds defined by ± 2 SD fall below zero for all studies, indicating that SA days cannot be appropriately meta-analyzed using mean and standard deviation summary statistics.

### Methodological data

#### Methodology ratings

The POMRS research methodology score had a median of 16.5 (IQR 12.0–20.5; range 8–26; possible range 0–44). See Additional file 3, Fig. 3 for item- and study-level ratings.

#### Risk-of-bias

For studies with RCT design, six of 17 (35.3%) were rated as having low risk of bias, while eight (50%) studies were rated “some concerns”, and two studies (12.5%) received a rating of high risk of bias. Regarding observational studies rated with the ROBINS-I tool, one study was rated as “critical”, five as “high” and one as having moderate risk of bias. See Additional file 3, Table 1 for overall risk of bias ratings. The RoB2 and ROBINS-I classifications are presented in Additional file 3, Tables 2 and 3.

### Meta-analysis

#### Attrition at post-treatment

The attrition rate for the individual studies is shown in Table 2. A meta-analysis of proportions showed a mean attrition rate of 9.7% (95% CI [6.7%, 13.8%], *k* = 25) with significant heterogeneity (*Q*(24) = 150, *p* < 0.0001, *I*^2^ = 84.0% [77.5%, 88.7%], τ^2^ = 0.54 [0.26; 1.79]). No moderators were statistically significant. A forest plot of attrition rates is shown in Additional file 3, Fig. 4.

#### Psychiatric symptoms

For within-group analyses, the mean pre-post effect size was *g* = 1.04. The pre to follow-up *g*-value was 1.09, indicating that the effect was maintained through follow-up, which had a median timespan of 8.5 months (*IQR* = 6–12, range 3–36 months). Complete results are reported in Table 3.

**Table 3:** Meta-analysis results of psychiatric symptoms

	k	*n*	g [95% CI]	SE	t	Q	I^2^% [95% CI]	τ^2^ between	τ^2^ within	ES prediction interval
Within group ES for psychiatric symptoms										
Post	21	49	1.04 [0.83, 1.25]	0.10	10.41***	325**	85.2 [81.2, 88.3]	0.13 [0.04, 0.34]	0.08 [0.04, 0.18]	[0.05, 2.04]
Follow-up	15	39	1.09 [0.87, 1.32]	0.11	10.31***	223**	82.9 [77.4, 87.0]	0.11 [0.03; 0.32]	0.07 [0.03; 0.15]	[0.16, 2.03]
Between groups ES for psychiatric symptoms										
Post	18	45	0.28 [0.10, 0.46]	0.09	-3.31**	159**	72.3 [62.7, 79.4]	0.11 [0.05, 0.25]	0.00 [0.00, 0.01]	[-0.43, 1.00]
Follow-up	15	39	0.11 [0.01, 0.20]	0.04	-2.45*	38	0.0 [0.0, 36.6]	0.01 [0.00, 0.06]	0.00 [0.00, 0.01]	[-0.15, 0.36]

* = <0.05, ** = <0.001, *** = <0.0001

##### Publication bias and sensitivity analyses

Egger’s test, adapted for multilevel meta-analysis, was not statistically significant, *p* = 0.307. The Bayesian random-effects meta-analysis indicated an unadjusted ES of 0.98 (95% CI [0.81, 1.15]) while the robust Bayesian model-averaged ES was 1.00 (95% CI [0.81, 1.27]), with an effect Bayes Factor (BF) at infinity (posterior probability = 1), and bias BF = 0.568 (posterior probability = 0.362). This indicates strong evidence for an effect and moderate evidence against bias.

For the within-group pre to follow-up data Egger’s test was not statistically significant, *p* = 0.469. The Bayesian random-effects meta-analysis indicated an unadjusted ES of 1.03 (95% CI [0.84, 1.21]) while the robust Bayesian model-averaged ES was 1.04 (95% CI [0.83, 1.32]), with an effect Bayes Factor (BF) at infinity (posterior probability = 1), and bias BF = 0.854 (posterior probability = 0.461). This indicates strong evidence for an effect and weak evidence against bias.

##### Moderator analyses

Given significant heterogeneity, moderator analyses were conducted for pre-post within-group comparisons. Higher scores on the CBT content subscale were associated with larger ES (*b* = 0.105, *SE* = 0.044, 95% CI [0.003, 0.206], *p* = 0.045), as were higher scores on the work-focused content subscale (*b* = 0.098, *SE* = 0.032, 95% CI [0.029, 0.168], *p* = 0.010). Other statistically significant moderators were number of sessions (*b* = 0.066, *SE* = 0.011, 95% CI [0.036, 0.097], *p* = 0.004), publication year (*b* = 0.034, *SE* = 0.013, 95% CI [0.001, 0.066], *p* = 0.046), and the categorical moderator for country. In a separate meta-regression model including all five moderators that were statistically significant on their own, the test for residual heterogeneity indicated that these moderators did not explain a substantial amount of heterogeneity of effect sizes (*QE*(df = 34) = 120, *p* < 0.0001).

The following moderators were not statistically significant: mean age, proportion of women, study design, POMRS score, sample size, presence of a workplace intervention, type of statistical analysis, mental disorder, setting, integration, therapist profession, and risk of bias rating.

**Fig. 4:**
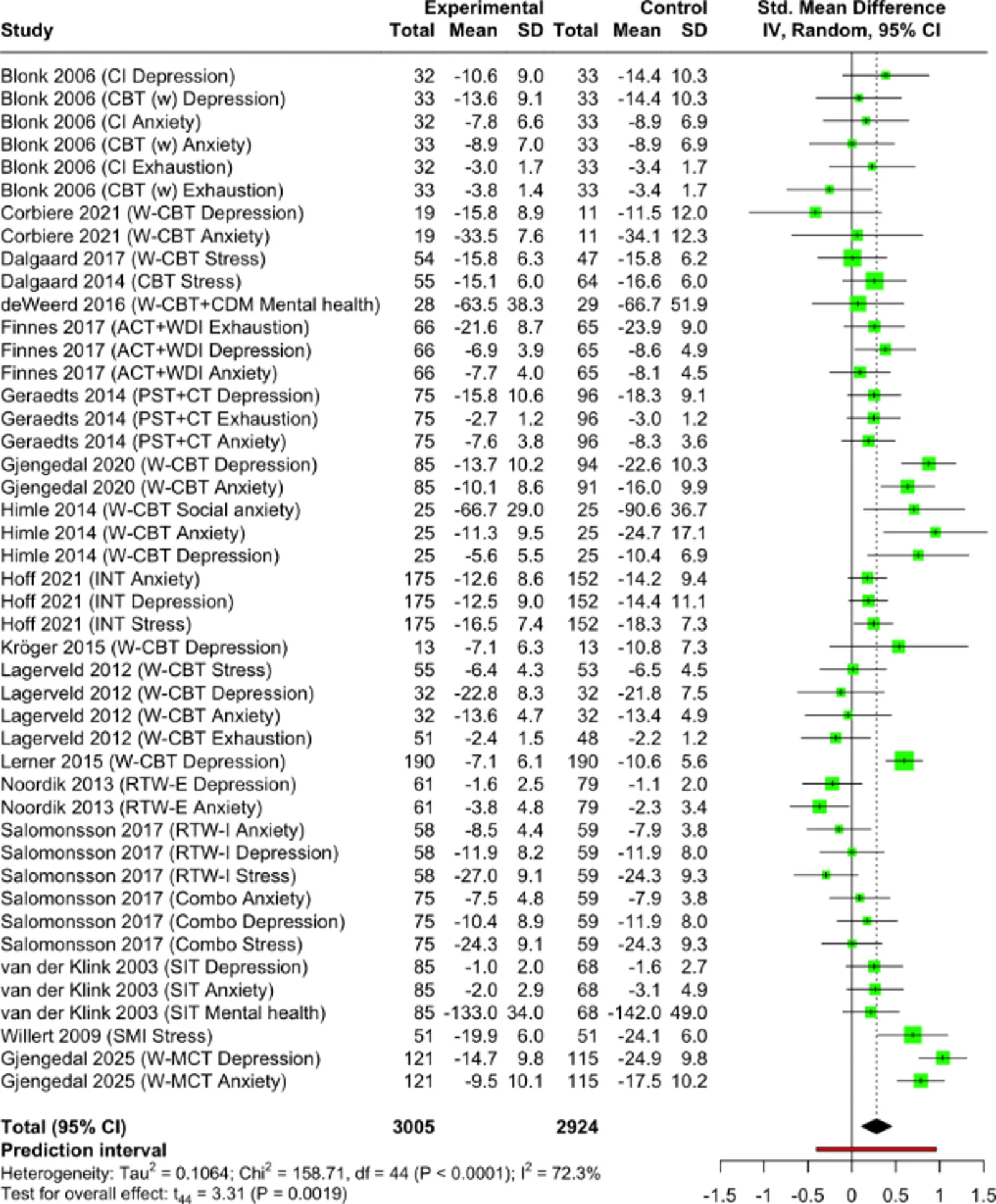
Forest plot for between-group symptom outcomes at post

For between-group analyses, there was a small mean ES at post, *g* = 0.28. The follow-up *g*-value was 0.11. See Fig. 4 for a forest plot of post-treatment results and Table 3 for all metrics.

##### Publication bias and sensitivity analyses

Egger’s test, adapted for multilevel meta-analysis, was not statistically significant, *p* = 0.349. The Bayesian random-effects meta-analysis indicated an unadjusted ES of 0.275 (95% CI [0.12, 0.43]) while the robust Bayesian model-averaged ES was 0.212 (95% CI [0.00, 0.42]), with an effect Bayes Factor (BF) = 4.133 (posterior probability = 0.805), and bias BF = 0.523 (posterior probability = 0.344). This indicates moderate evidence for an effect and against bias. The Z-curve plot is shown in Additional file 3, Fig. 5.

For the follow-up data, Egger’s test was not statistically significant (*p* = 0.641). The Bayesian random-effects model resulted in an unadjusted ES of 0.105 (95% CI [0.03, 0.19]) while the robust Bayesian model-averaged ES was 0.04 (95% CI [0.00, 0.15]), with an effect Bayes Factor (BF) = 0.557 (posterior probability = 0.358), and bias BF = 1.510 (posterior probability = 0.602). This indicates moderate evidence against an effect and weak evidence for bias.

##### Moderator analyses

Only two moderators were shown to be statistically significant in the pre-post between-group analyses: country (*p* < 0.001) and comparison group (*p* = 0.007). No statistically significant residual heterogeneity remained in the model with country as a moderator (*QE*(38) = 45.8, *p* = 0.182), in contrast to the model with comparison as moderator (*QE*(42) = 66.4, *p* = 0.010). The only comparison group showing a statistically significant effect was WLC (*b* = 0.77, *SE* = 0.098, 95% CI [0.51, 1.04], *p* = 0.001), with TAU at *b* = 0.19 (95% CI [-0.13, 0.51]) and other CBT treatments at *b* = 0.03 (95% CI [-0.25, 0.31]).

The following moderators were not statistically significant: CBT subscale, work-focused subscale, publication year, age, proportion of women, study design, workplace intervention, type of statistical analysis, POMRS score, mental disorder, number of treatment sessions, setting, degree of integration, therapist profession, treatment format, and risk of bias rating.

#### Work outcomes

**Fig. 5:**
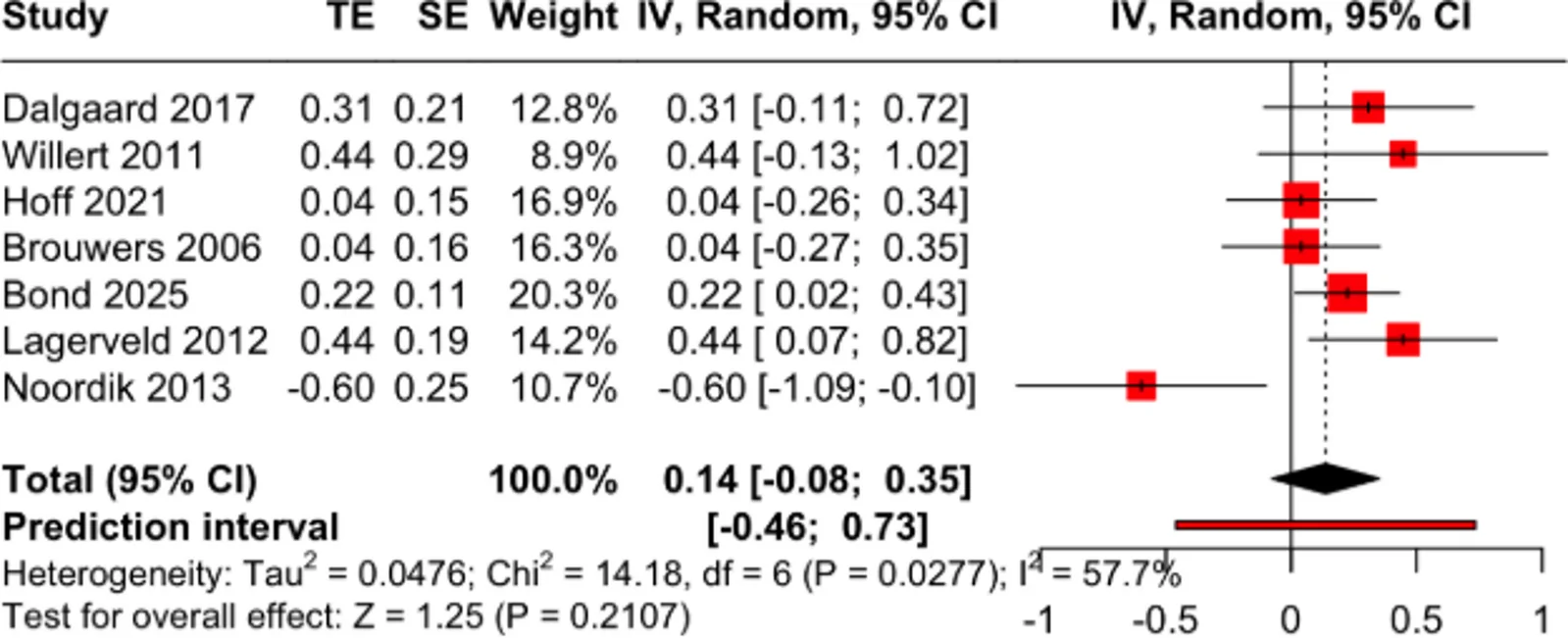
Forest plot for meta-analysis of Time to full return to work in studies reporting hazard ratios

Seven studies reported within-group hazard ratios (HRs) from Cox proportional hazards models for time to full RTW. Follow-up durations were generally approximately one year: three studies used 52 weeks, one 44 weeks, one 48 weeks, one 78 weeks, and one a three-year follow-up. The meta-analytic effect was *HR* = 1.15 (95% *CI* [0.93, 1.42], *k* = 7, log *HR* = 0.14 [-0.08, 0.35]), which was moderately heterogeneous (*Q*(6) = 14.18, *p* = 0.028, *I*^2^ = 57.7% [2.0%; 81.7%], τ^2^ = 0.05 [0.00; 0.57]) with ES prediction interval [-0.46, 0.73]. Figure 5 illustrates the results in a forest plot. As no time point included 10 or more studies, analyses of publication bias and moderators were not conducted.

For working status, within-group analyses showed that the proportion of participants reporting full RTW at post was 0.56 (95% CI [0.43; 0.69], *k* = 11), which was significantly heterogeneous (*Q*(10) = 68.78,1.69 [0.49; 14.25]) with ES prediction interval *p* < 0.0001, *I*^2^ = 85.5% [75.7%; 91.3%], τ^2^ = 0.38 [0.17; 3.30]) with ES prediction interval [0.23, 0.84].

**Fig. 6:**
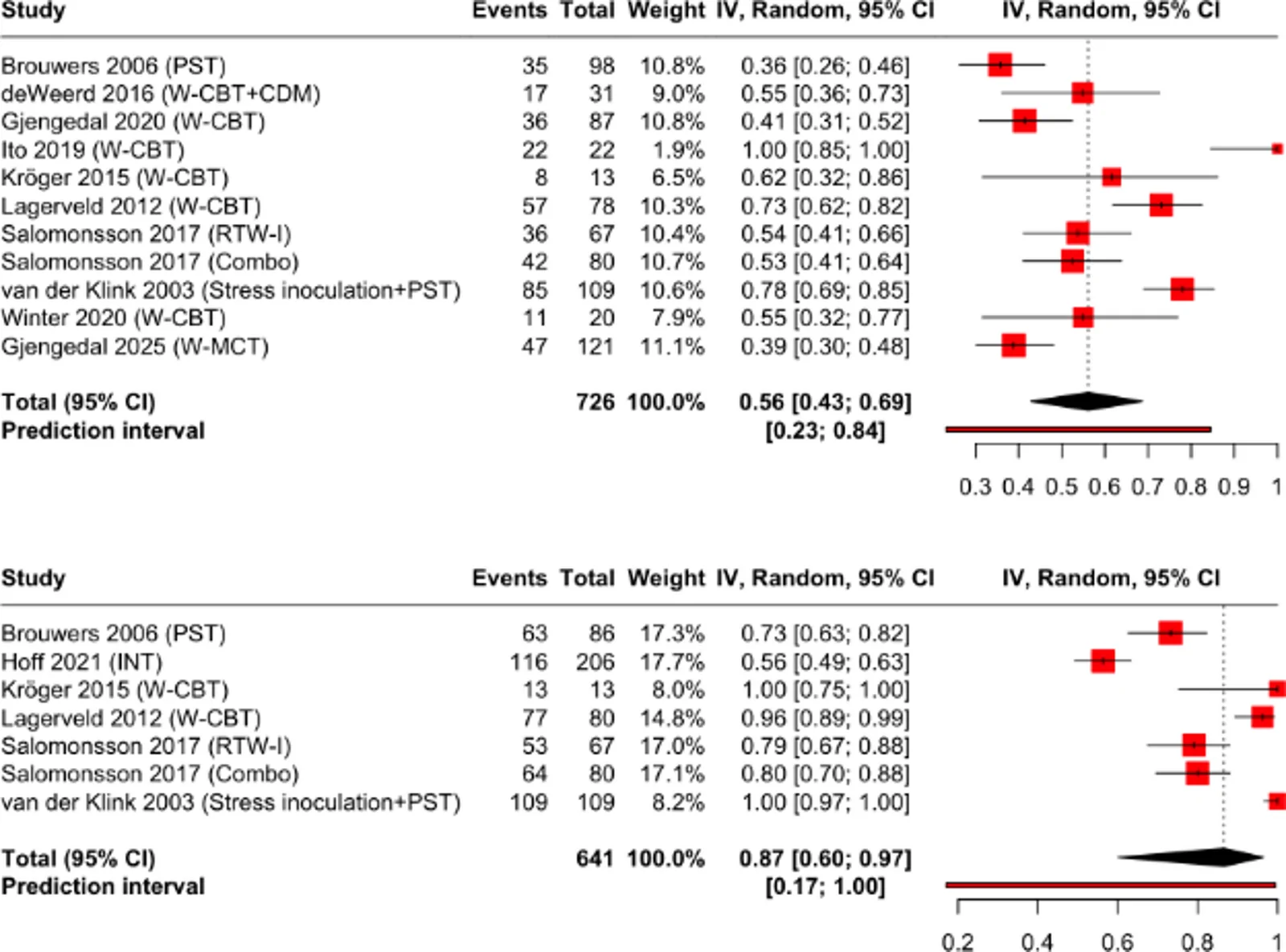
Forest plots for proportion of full return to work within group at post (upper plot) and follow-up (lower plot)

At follow-up, the proportion of full RTW was 0.87 (95% CI [0.60; 0.97], k = 7), which was significantly heterogeneous (*Q*(6) = 54.12, *p* < 0.0001, *I*^2^ = 88.9% [79.6%; 94.0%], τ^2^ = 1.69 [0.49; 14.25]) with ES prediction interval [0.17, 1.00]. Figure 6 contains forest plots for post and follow-up results.

Peters’ test for funnel plot asymmetry was not statistically significant for the within-group post-treatment analysis (*t*(8) = 0.37, *p* = 0.718, bias estimate = 6.8, *SE* = 18.2), indicating no evidence of publication bias. This analysis included 11 studies, permitting moderator analyses; however, heterogeneity was high (*I²* = 85.5%). None of the examined moderators were statistically significant.

Between-group analyses for working status yielded a log risk ratio (RR) for full RTW at post of 0.21 (95% CI [0.05; 0.37], *RR* = 1.23 [1.05, 1.45], *k* = 9, *z* = 2.59, *p* = 0.010), which was significantly heterogeneous (*Q*(8) = 295.46, *p* < 0.0001, *I*^2^ = 97.3% [96.2, 98.1], τ^2^ = 0.06 [0.02, 0.21]) with ES prediction interval [-0.37, 0.79].

**Fig. 7:**
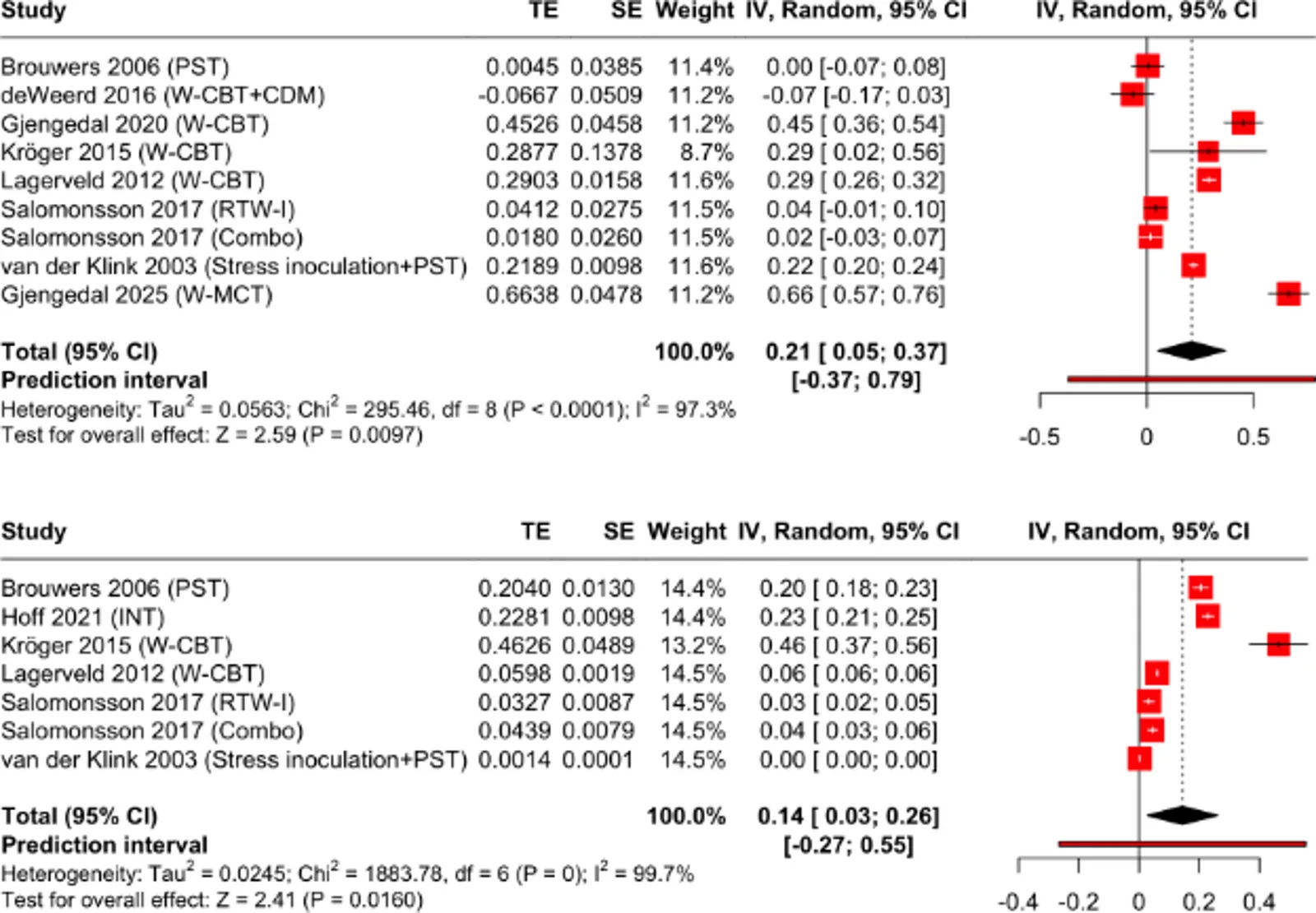
Forest plots for meta-analysis of between group differences of full return to work at post (upper plot) and follow-up (lower plot)

At follow-up, the log risk ratio was 0.14 (95% CI [0.03; 0.26], *RR* = 1.15 [1.03, 1.30], *k* = 7, *z* = 2.41, *p* = 0.016), which was significantly heterogeneous (*Q*(6) = 1883.78, *p* < 0.0001, *I*^2^ = 99.7% [99.6, 99.7], τ^2^ = 0.02 [0.01, 0.13]) with ES prediction interval [-0.27, 0.55]. Results are illustrated in Fig. 7. Since no time point included 10 or more studies, analyses of publication bias and moderators were not conducted.

For self-assessed work functioning, within-group analyses showed that the mean pre-post within-group effect size was *g* = 0.97 (95% CI [0.48; 1.46], *k* = 6, *t*(5) = 5.10, *p* = 0.004), which was significantly heterogeneous (*Q*(5) = 23.12, *p* = 0.0003, *I*^2^ = 78.4% [52.4%; 90.2%], τ^2^ = 0.16 [0.03; 1.33]) with ES prediction interval [-0.15, 2.10].

**Fig. 8:**
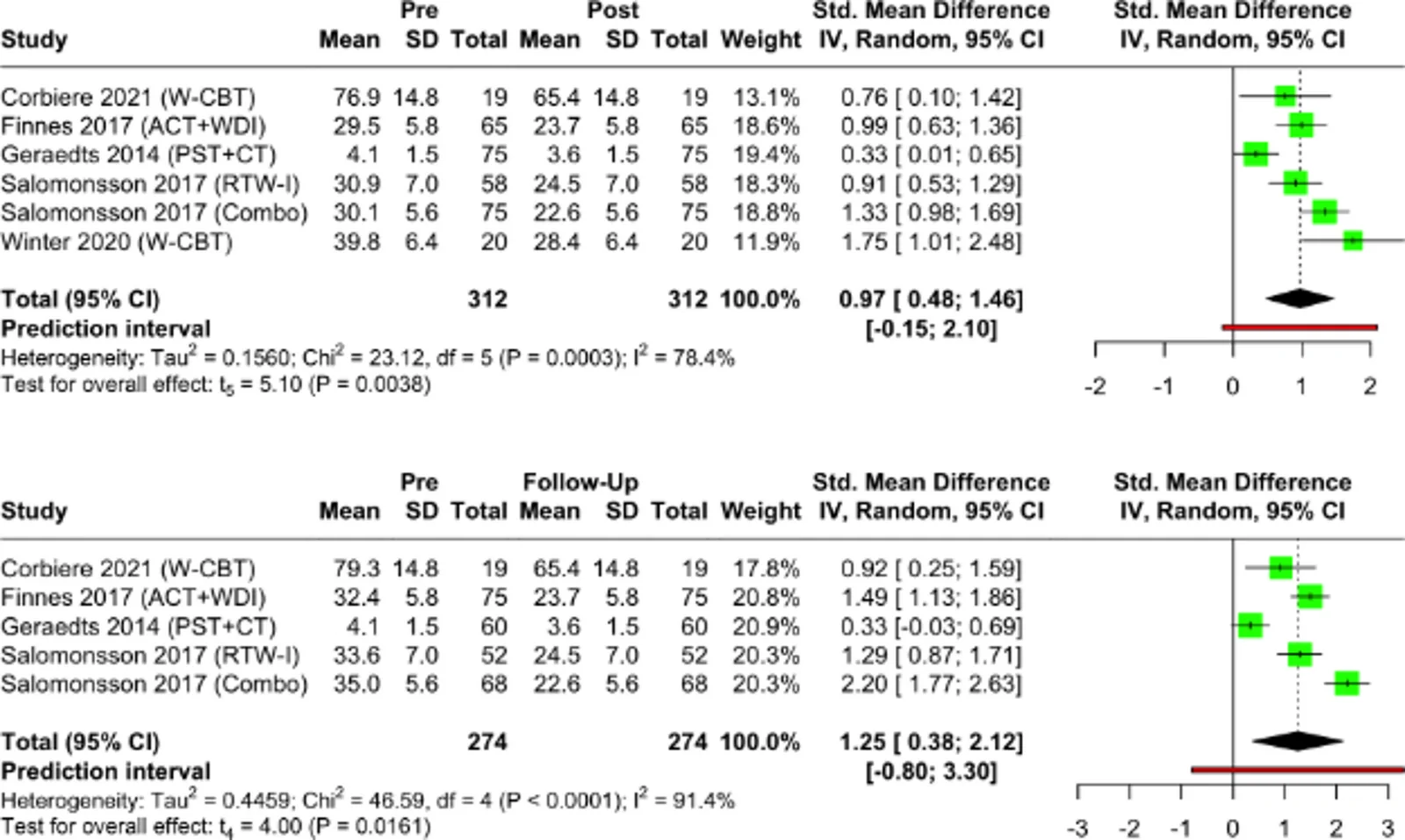
Forest plots of self-reported work functioning within-group, pre to post (upper plot) and pre to follow-up (lower plot)

The pre to follow-up *g*-value was 1.25 (95% CI [0.38; 2.12], *k* = 5, *t*(4) = 4.00, *p* = 0.016), which was significantly heterogeneous (*Q*(4) = 46.59, *p* < 0.0001, *I*^2^ = 91.4% [82.9%; 95.7%], τ^2^ = 0.45 [0.13; 3.92]) with ES prediction interval [-0.80, 3.30]. Results are illustrated in Fig. 8. The somewhat larger effect size for the follow-up period compared to post potentially indicates that the treatment effect (or effect of time) on work outcomes may be delayed compared with symptom outcomes. Work functioning had too few studies to conduct publication bias or moderator analyses.

**Fig. 9:**
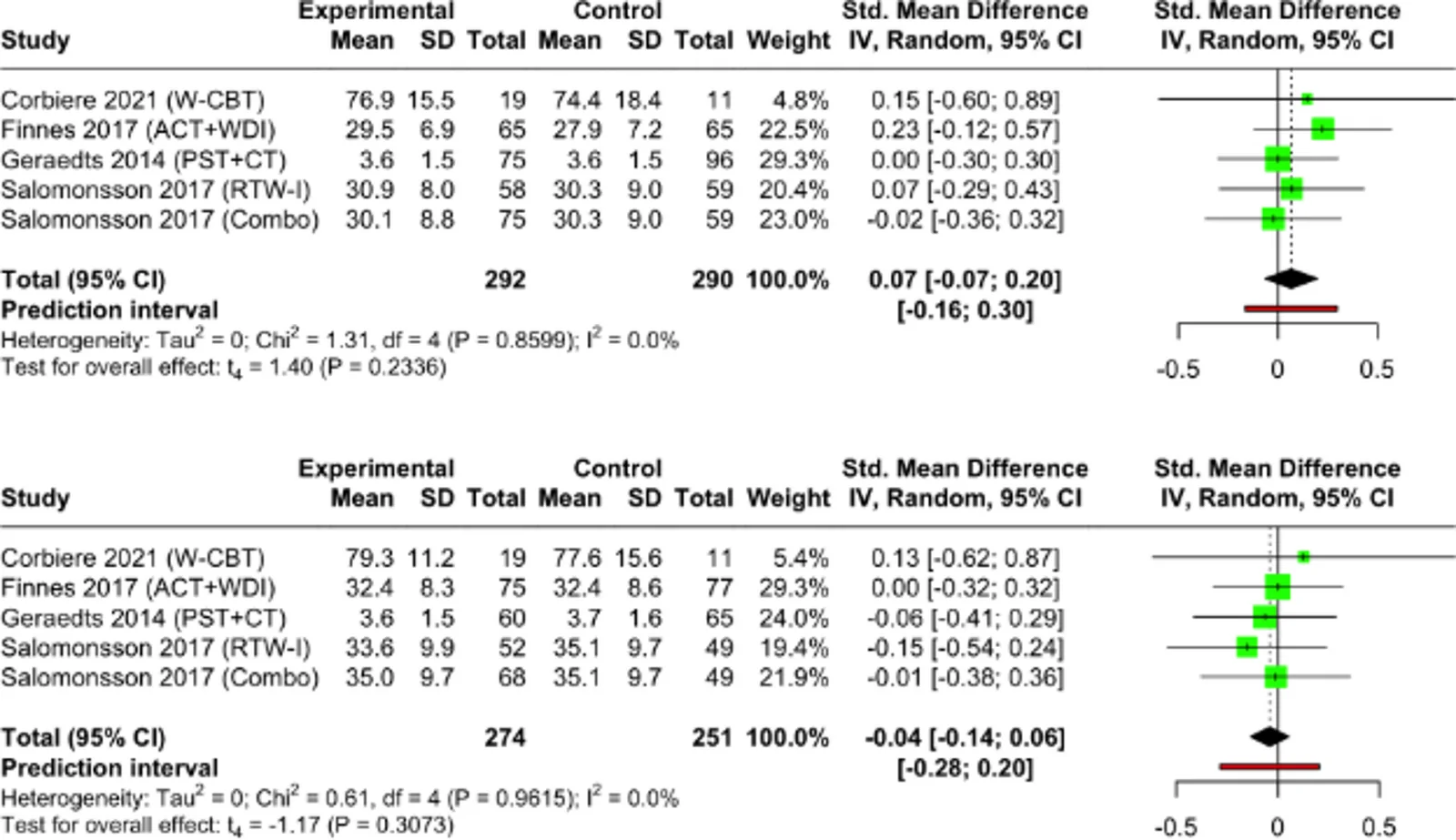
Forest plots of self-reported work functioning between-group at post (upper plot) and follow-up (lower plot)

Between-group analyses for self-reported work functioning showed that the mean effect size at post was *g* = 0.07 [-0.07; 0.20] (*k* = 6, *t*(4) = 1.40, *p* = 0.234) and at follow-up, *g* = -0.04 [-0.14; 0.06] (*k* = 5, *t*(4) = -1.17, *p* = 0.307). There was no significant heterogeneity at either time point, with post *Q*(4) = 1.31, *p* = 0.860, *I*^2^ = 0.0% [0.0%; 79.2%], τ^2^ = 0 [0.00; 0.05], ES prediction interval [-0.16, 0.30]; and follow-up *Q*(4) = 0.61, *p* = 0.9615, *I*^2^ = 0.0% [0.0%; 79.2%], τ^2^ = 0 [0.00; 0.01], ES prediction interval [-0.28, 0.20], respectively. Results are illustrated in Fig. 9.

## Discussion

This systematic review and meta-analysis evaluated the evidence for work-focused cognitive behavioural therapy (W-CBT) among individuals on sickness absence due to common mental disorders, examined moderators of treatment effects, assessed methodological quality, and identified core intervention components. Overall, the findings indicate that W-CBT is associated with large improvements in psychiatric symptoms within treatment groups and modest but statistically significant advantages over control conditions in between-group comparisons. For work-related outcomes, substantial proportions of participants returned to work following W-CBT, and relative RTW rates were higher compared with control conditions.

### Effects on psychiatric symptoms

Large within-group effects were observed for psychiatric symptom reduction from pre- to post-treatment and were maintained through follow-up, although they were not as large as those typically reported in standard CBT trials for depression and anxiety [70, 71]. These findings suggest that W-CBT interventions are clinically potent for symptom reduction in working populations with CMDs. While W-CBT generates lower ESs compared with standard CBT, these studies are also characterized by lower methodological scores. Öst et al. reported mean POMRS scores in effectiveness trials for depression and anxiety disorders of 22.7 and 22.3, respectively [70, 71]. In the present review, the median POMRS score was 16.5, suggesting less methodological rigour compared with standard CBT trials. W-CBT studies also typically address symptoms from a transdiagnostic perspective, which does not allow for disorder-specific treatment. Between-group effects were smaller, particularly when W-CBT was compared with active control conditions, which is consistent with the broader psychotherapy literature [72] and expected when comparing interventions that share common therapeutic elements. Importantly, the absence of large between-group differences compared with other CBT formats should not be interpreted as a lack of effectiveness, but rather as evidence that incorporating work-focused components does not compromise, and may enhance, the effectiveness of CBT for symptom outcomes.

### Work-related outcomes and delayed functional recovery

Across studies, approximately 56% of participants had returned to work at post-treatment and 87% at follow-up, indicating substantial functional recovery over time. Between-group analyses showed a significantly increased relative probability of full RTW in W-CBT compared with control conditions at both post-treatment and follow-up. These findings align with the theoretical premise of W-CBT, in which work participation is conceptualized as both a treatment goal and a therapeutic context.

Notably, work functioning outcomes demonstrated large within-group effects that were more pronounced at follow-up than at post-treatment. This temporal pattern suggests that improvements in work functioning may lag behind symptom reduction, consistent with prior evidence that functional recovery often follows clinical remission rather than occurring concurrently [73]. The lack of significant between-group effects for work functioning likely reflects limited statistical power, heterogeneity in measurement instruments, and the relatively small number of studies reporting this outcome.

### Moderators of treatment effects and intervention content

Moderator analyses provided partial support for the conceptual foundation of W-CBT. For within-group symptom outcomes, higher scores on both the CBT content subscale and the work-focused content subscale were associated with larger effect sizes, indicating that interventions incorporating core CBT procedures alongside explicit work-related strategies yield stronger symptom improvements. The number of treatment sessions emerged as a significant moderator, suggesting a dose–response relationship in which more intensive treatments produce greater gains. Publication year was also positively associated with effect sizes, potentially reflecting the maturation of W-CBT protocols in more recent trials. Country-level differences were also significant, which may reflect variation in social insurance systems, labour market policies, or cultural factors influencing sickness absence and return to work. However, when all five significant univariable moderators were entered simultaneously, substantial residual heterogeneity remained and most moderators were no longer significant. This should be interpreted cautiously, as the analysis was based on a relatively small number of studies, but also potentially indicates a complex relationship among these moderator variables.

For between-group symptom analyses, only country and type of comparison condition emerged as significant moderators. Significant effects were observed only relative to waitlist control conditions, whereas comparisons with treatment as usual and alternative CBT-based interventions yielded non-significant differences. This pattern is consistent with the broader psychotherapy literature, where effect sizes diminish as the stringency of the comparison condition increases. Notably, the moderating effect of country and comparison condition may be partially confounded, as two of the three Norwegian studies employed a waitlist control design. It is therefore difficult to disentangle whether the country-level differences reflect genuine contextual variation, such as differences in usual care quality or social insurance systems, or are primarily driven by the choice of comparison condition. That country explained residual heterogeneity in between-group but not within-group analyses is consistent with the latter interpretation, as the type of control condition would be expected to influence relative but not absolute treatment effects.

In contrast, therapist profession, treatment format, study setting, mental disorder, workplace intervention, study design, type of statistical analysis, methodological quality score, and risk of bias rating were not significant moderators in either within- or between-group analyses. These null findings are encouraging from an implementation perspective, as they suggest that W-CBT may be flexibly delivered across professional groups, settings, and formats without substantial loss of effectiveness. The comparability of effect sizes across randomized and non-randomized designs, and across intention-to-treat and completer analyses, further strengthens the consistency of the findings since it suggests the pooled estimates are not being driven by design differences or by how attrition was handled.

### Methodological heterogeneity and implications for RTW research

A central contribution of this review is the examination of how RTW outcomes are measured and analyzed. Core outcome sets for RTW have recently been proposed, including the proportion of individuals returning to work after sickness absence and time to RTW [74]. These outcomes are already frequently reported in the literature; however, important methodological challenges remain, particularly regarding how RTW data are analyzed and reported in primary studies.

Consistent with previous reviews, we found substantial heterogeneity in RTW outcome definitions, time frames, and statistical approaches, which complicates both interpretation and evidence synthesis [15, 17, 75, 76]. While Shiri et al. [75] attribute mixed findings primarily to limited power or true null effects, our review highlights that statistical methodological issues may play a central role. Many studies relied on summary statistics assuming normally distributed data for outcomes that are inherently bounded, zero- and/or one-inflated, and skewed, such as days on sickness absence or time to RTW. In several cases, reported means and standard deviations implied impossible values (e.g., negative days), highlighting a fundamental mismatch between data properties, summary statistics, and statistical methods used.

These methodological issues are not merely technical but have substantive implications for inference. Analyzing heavily skewed and/or bounded outcomes with unbounded linear models (e.g., ordinary least squares regression) may result in invalid point estimates, confidence intervals, and p-values, and can yield implausible predictions, such as negative values for strictly positive outcomes like time to RTW. Consistent with these concerns, previous reviews have reported difficulties in conducting meta-analyses of RTW outcomes due to “contradictory findings and wide confidence intervals” [17], which are likely partly the consequence of inappropriate summary statistics and modeling choices in the primary studies. Although advanced statistical models are available, such as ordered beta regression [77, 78] when data are bounded and a substantial proportion of observations fall at the bounds [79], their outputs are difficult to integrate into conventional meta-analytic frameworks without access to individual participant data. This limitation underscores the need for greater methodological alignment between primary studies and evidence synthesis efforts.

Data-sharing practices are also important to consider, not least since meta-analysis using individual participant data is considered the gold standard [80, 81]. None of the studies included in this review had openly shared data, and it is well known that the ubiquitous data availability statement that “data will be shared upon reasonable request” is seldom a reality [82]. With access to individual participant data, the issues with meta-analysis caused by different measures used can also be handled more appropriately [81].

#### Use of hazard ratios in RTW meta-analysis

Time-to-event outcomes were commonly analyzed using Cox proportional hazards models, and hazard ratios (HRs) were included in the present meta-analysis. However, HRs are non-collapsible [83], have built-in selection bias [84], are sensitive to effect modification by covariates [85], and are dependent on follow-up duration, which complicates their comparability across studies [68]. These issues have relevance for evidence synthesis since the comparability of HR estimates and SEs across studies may be questionable, making meta-analysis potentially problematic. A possible solution for the effect modification issue is to use inverse probability of treatment weighting to include covariates in the analysis without changing the interpretation of the HR [86]. The issues mentioned constitute good reasons to consider other statistical models or coefficients for time to RTW data, such as risk differences or risk ratios [87].

### Strengths and limitations

This review has several strengths. It applies advanced meta-analytic techniques to address statistical dependence among multiple outcomes, using multilevel models and cluster-robust standard errors for confidence intervals to account for dependence between multiple effect sizes from the same study. Moderator analyses were conducted in line with current methodological recommendations, rather than evaluating moderators only one by one [88]. We also used meta-regression in conjunction with risk of bias tools to explore whether and how effect sizes vary systematically with study quality. Publication bias was examined using both frequentist and Bayesian approaches, providing convergent evidence for the robustness of the main findings.

Limitations should also be noted. Only studies published in English-language journals were included, and results from unpublished studies or studies published in other languages may differ. Uncontrolled pre–post effect sizes are susceptible to non-specific effects such as spontaneous recovery and regression to the mean. High levels of heterogeneity across studies limit the precision of pooled estimates and reduce the power of some bias-detection methods, rendering techniques such as trim-and-fill inappropriate. Finally, the limited number of studies reporting work-related outcomes constrained moderator and sensitivity analyses as well as statistical power.

### Implications for research and practice

Collectively, the findings highlight two key opportunities for advancing the field. First, methodological improvements are needed in the design, analysis, and reporting of RTW outcomes. While complete standardization may be neither feasible nor desirable, greater transparency, appropriate statistical modeling, and alignment with emerging core outcome sets would substantially strengthen the evidence base. Second, the results support continued development and evaluation of interventions that integrate mental health treatment with work-focused strategies. W-CBT represents a promising approach that aligns clinical recovery with functional and societal outcomes, which is central to public health efforts aimed at reducing work disability due to CMDs.

### Public health implications

In European welfare states, common mental disorders constitute one of the leading causes of sickness absence and long-term work disability [89], placing sustained pressure on healthcare systems, social insurance schemes, and labour markets. Interventions that address both symptom reduction and work participation are therefore of central public health relevance. The findings of this review suggest that W-CBT can contribute to these dual objectives by improving mental health outcomes while increasing the likelihood of RTW.

From a public health systems perspective, W-CBT aligns well with the organization of primary care and occupational health services in many European countries, where mental health treatment, sickness certification, and RTW coordination often intersect. The observation that W-CBT performs well for symptom reduction, while offering additional benefits for work participation, supports its use as an early and integrated intervention during sickness absence. Earlier RTW may reduce the risk of prolonged absence, recurrent sick leave, and permanent labour market exclusion, outcomes that are strongly associated with long-term social and economic disadvantage.

The results also suggest that W-CBT is potentially scalable within existing healthcare infrastructures. Effects were observed across different countries, settings, and therapist professions, indicating that specialized vocational services are not a prerequisite for effectiveness. However, the substantial heterogeneity and methodological weaknesses identified in RTW outcome measurement highlight a critical public health research gap. Improving analytic approaches and harmonizing outcome reporting in line with emerging core outcome sets will be essential for translating effective interventions such as W-CBT into policy, clinical guidelines, and large-scale implementation.

## Conclusions

Work-focused cognitive behavioural therapy is associated with substantial improvements in psychiatric symptoms and meaningful gains in work participation among individuals on sickness absence due to common mental disorders. Interventions that integrate core CBT techniques with explicit work-focused components appear particularly beneficial. Future research should prioritize methodological rigour in RTW outcome analysis and continue to refine and test integrated intervention models that address both mental health and work functioning in parallel.

## Supplementary Information

Below is the link to the electronic supplementary material.

Supplementary Material 1

Supplementary Material 2

Supplementary Material 3

Supplementary Material 4

## Data Availability

All extracted data and the R analysis code needed to reproduce the results is available at: https://osf.io/6mr7v/files/osfstorage.

## Abbreviations

AD: Adjustment Disorder
BDI: Beck Depression Inventory
BF: Bayes Factor
BT: Behavioural Therapy
CBT: Cognitive Behavioural Therapy
CES-D: Center for Epidemiologic Studies Depression Scale
CI: Confidence Interval
CMD: Common Mental Disorder
CR2: Cluster—Robust variance estimator (type 2)
CT: Cognitive Therapy
DASS: Depression Anxiety Stress Scale
ES: Effect Size
EU: European Union
EWPS: Endicott Work Productivity Scale
GAD-7: Generalized Anxiety Disorder-7
HPQ: (WHO) Health and Work Performance Questionnaire
HR: Hazard Ratio
ICC: Intraclass Correlation CoefficiOccupational Health Servicesent
IQR: Interquartile Range
ITT: Intention-to-Treat
MADRS: Montgomery Åsberg Depression Rating Scale
MeSH: Medical Subject Headings
OECD: Organisation for Economic Co-operation and Development
OHS: Occupational Health Services
PHQ-9: Patient Health Questionnaire-9
PICOS: Population, Intervention, Comparison, Outcome, Study design
POMRS: Psychotherapy Outcome Study Methodology Rating Scale
PRISMA: Preferred Reporting Items for Systematic Reviews and Meta-Analyses
PROSPERO: International Prospective Register of Systematic Reviews
QE: Q-statistic for residual heterogeneity
RCT: Randomized Controlled Trial
REML: Restricted Maximum Likelihood (Estimator)
RoB2: Cochrane Risk of Bias tool, version 2
RoBMA: Robust Bayesian Meta-Analysis
ROBINS-I: Risk Of Bias In Non-randomised Studies of Interventions
RR: Risk Ratio
RTW: Return-to-Work
SA: Sickness Absence
SAD: Social Anxiety Disorder
SBU: Statens beredning för medicinsk och social utvärdering (Swedish Council on Health Technology Assessment)
SD: Standard Deviation
SE: Standard Error
SMD: Standardized Mean Difference
TAU: Treatment as Usual
USA: United States of America
W-CBT: Work-focused Cognitive Behavioural Therapy
WLC: Waitlist Control
