# Supplementary material for: Work-focused cognitive behavioural therapy for common mental disorders: a systematic review and meta-analysis
Source: BMC Psychol. 2026 Sep 22;14:1374. doi: 10.1186/s40359-026-05648-2 (PMC13599331; doi:10.1186/s40359-026-05648-2)
Supplement: Supplementary file 1 [file 40359_2026_5648_MOESM1_ESM.docx]

**S2. Included articles**

Blonk, R. W. B., Brenninkmeijer, V., Lagerveld, S. E., & Houtman, I. L. D. (2006). Return to work: A comparison of two cognitive behavioural interventions in cases of work-related psychological complaints among the self-employed. *Work & Stress*, *20*(2), 129–144. <https://doi.org/10.1080/02678370600856615>

Bond, C. B., Willert, M. V., Ditlevsen, D. N., Sandal, L. F., & Brandt, L. (2025). Effect of Cognitive Behavioural Stress Management on Return-to-Work Amongst Sick-Listed Employees. *Journal of Occupational Rehabilitation*. <https://doi.org/10.1007/s10926-025-10306-2>

Brouwers, E. P. M., Tiemens, B. G., Terluin, B., & Verhaak, P. F. M. (2006). Effectiveness of an intervention to reduce sickness absence in patients with emotional distress or minor mental disorders: A randomized controlled effectiveness trial. *General Hospital Psychiatry*, *28*(3), 223–229. <https://doi.org/10.1016/j.genhosppsych.2006.02.005>

Corbière, M., Lachance, J.-P., Jean-Baptiste, F., Hache-Labelle, C., Riopel, G., & Lecomte, T. (2022). Healthy Minds: Group Cognitive-Behavioral Intervention for Sustainable Return to Work After a Sick Leave Due to Depression. *Journal of Occupational Rehabilitation*, *32*(3), 505–514. <https://doi.org/10.1007/s10926-021-09991-6>

Dalgaard, L., Eskildsen, A., Carstensen, O., Willert, M. V., Andersen, J. H., & Glasscock, D. J. (2014). Changes in self-reported sleep and cognitive failures: A randomized controlled trial of a stress management intervention. *Scandinavian Journal of Work, Environment & Health*, *40*(6), 569–581. <https://doi.org/10.5271/sjweh.3460>

Dalgaard, V. L., Andersen, L. P. S., Andersen, J. H., Willert, M. V., Carstensen, O., & Glasscock, D. J. (2017). Work-focused cognitive behavioral intervention for psychological complaints in patients on sick leave due to work-related stress: Results from a randomized controlled trial. *Journal of Negative Results in BioMedicine*, *16*(1), 13. <https://doi.org/10.1186/s12952-017-0078-z>

Dalgaard, V. L., Aschbacher, K., Andersen, J. H., Glasscock, D. J., Willert, M. V., Carstensen, O., & Biering, K. (2017). Return to work after work-related stress: A randomized controlled trial of a work-focused cognitive behavioral intervention. *Scandinavian Journal of Work, Environment & Health*, *43*(5), 436–446. <https://doi.org/10.5271/sjweh.3655>

De Weerd, B. J., Van Dijk, M. K., Van Der Linden, J. N., Roelen, C. A. M., & Verbraak, M. J. P. M. (2016). The effectiveness of a convergence dialogue meeting with the employer in promoting return to work as part of the cognitive-behavioural treatment of common mental disorders: A randomized controlled trial. *Work*, *54*(3), 647–655. <https://doi.org/10.3233/WOR-162307>

Finnes, A., Ghaderi, A., Dahl, J., Nager, A., & Enebrink, P. (2019). Randomized controlled trial of acceptance and commitment therapy and a workplace intervention for sickness absence due to mental disorders. *Journal of Occupational Health Psychology*, *24*(1), 198–212. <https://doi.org/10.1037/ocp0000097>

Geraedts, A. S., Kleiboer, A. M., Twisk, J., Wiezer, N. M., Van Mechelen, W., & Cuijpers, P. (2014). Long-Term Results of a Web-Based Guided Self-Help Intervention for Employees With Depressive Symptoms: Randomized Controlled Trial. *Journal of Medical Internet Research*, *16*(7), e168. <https://doi.org/10.2196/jmir.3539>

Gjengedal, R. G. H., Hannisdal, M., Osnes, K., Reme, S. E., Wells, A., Blonk, R., Lending, H. D., Johnson, S. U., Lagerveld, S. E., Anyan, F., Nordahl, H. M., Gerritsen, R. B. T. W., Bjørndal, M. T., Wright, D., Sandin, K., Bjøntegård, K. S., Schwach, J., & Hjemdal, O. (2025). Metacognitive therapy and work-focus for patients with depression, anxiety or comorbid depression and anxiety on sick leave: A single-centre, open-label randomised controlled trial. *eClinicalMedicine*, *89*. <https://doi.org/10.1016/j.eclinm.2025.103613>

Gjengedal, R. G. H., Reme, S. E., Osnes, K., Lagerfeld, S. E., Blonk, R. W. B., Sandin, K., Berge, T., & Hjemdal, O. (2020). Work-focused therapy for common mental disorders: A naturalistic study comparing an intervention group with a waitlist control group. *Work*, *66*(3), 657–667. <https://doi.org/10.3233/WOR-203208>

Himle, J. A., Bybee, D., Steinberger, E., Laviolette, W. T., Weaver, A., Vlnka, S., Golenberg, Z., Levine, D. S., Heimberg, R. G., & O’Donnell, L. A. (2014). Work-related CBT versus vocational services as usual for unemployed persons with social anxiety disorder: A randomized controlled pilot trial. *Behaviour Research and Therapy*, *63*, 169–176. <https://doi.org/10.1016/j.brat.2014.10.005>

Ito, D., Okabe, Y., Nobushige, A., Saito, S., & Takahashi, Y. (2025). Functional assertiveness with acceptance and commitment therapy for employees returning to work: A preliminary investigation. *Frontiers in Psychology*, *16*, 1415212. <https://doi.org/10.3389/fpsyg.2025.1415212>

Kroger, C., Bode, K., Wunsch, E. M., Kliem, S., Grocholewski, A., & Finger, F. (2015). Work-related treatment for major depressive disorder and incapacity to work: Preliminary findings of a controlled, matched study. *Journal of Occupational Health Psychology*, *20*(2), 248–258. <https://doi.org/10.1037/a0038341>

Lagerveld, S. E., Blonk, R. W., Brenninkmeijer, V., Wijngaards-de Meij, L., & Schaufeli, W. B. (2012). Work-focused treatment of common mental disorders and return to work: A comparative outcome study. *Journal of Occupational Health Psychology*, *17*(2), 220–234. <https://doi.org/10.1037/a0027049>

Lerner, D., Adler, D. A., Rogers, W. H., Chang, H., Greenhill, A., Cymerman, E., & Azocar, F. (2015). A Randomized Clinical Trial of a Telephone Depression Intervention to Reduce Employee Presenteeism and Absenteeism. *Psychiatric Services*, *66*(6), 570–577. <https://doi.org/10.1176/appi.ps.201400350>

Monsen, H., Vøllestad, J., Prescott, P., Røren, A., Bruvik, K., Berge, T., Wallace, P. W., Nordgreen, T., Titov, N., & Hovland, A. (2025). Work-focused therapy in an internet-based CBT format – A mixed methods feasibility study. *Internet Interventions*, *41*, 100855. <https://doi.org/10.1016/j.invent.2025.100855>

Noordik, E., Van Der Klink, J. J., Geskus, R. B., De Boer, M. R., Van Dijk, F. J., & Nieuwenhuijsen, K. (2013). Effectiveness of an exposure-based return-to-work program for workers on sick leave due to common mental disorders: A cluster-randomized controlled trial. *Scandinavian Journal of Work, Environment & Health*, *39*(2), 144–154. <https://doi.org/10.5271/sjweh.3320>

Reme, S. E., Grasdal, A. L., Lovvik, C., Lie, S. A., & Overland, S. (2015). Work-focused cognitive-behavioural therapy and individual job support to increase work participation in common mental disorders: A randomised controlled multicentre trial. *Occupational and Environmental Medicine*, *72*(10), 745–752. <https://doi.org/10.1136/oemed-2014-102700>

Salomonsson, S., Santoft, F., Lindsater, E., Ejeby, K., Ljotsson, B., Ost, L. G., Ingvar, M., Lekander, M., & Hedman-Lagerlof, E. (2017). Cognitive-behavioural therapy and return-to-work intervention for patients on sick leave due to common mental disorders: A randomised controlled trial. *Occupational and Environmental Medicine*, *74*(12), 905–912. <https://doi.org/10.1136/oemed-2017-104342>

van der Klink, J. J., Blonk, R. W., Schene, A. H., & van Dijk, F. J. (2003). Reducing long term sickness absence by an activating intervention in adjustment disorders: A cluster randomised controlled design. *Occupational and Environmental Medicine*, *60*(6), 429–437.

Willert, M. V., Thulstrup, A. M., Hertz, J., & Bonde, J. P. (2009). Changes in stress and coping from a randomized controlled trial of a three-month stress management intervention. *Scandinavian Journal of Work, Environment & Health*, *35*(2), 145–152. <https://doi.org/10.5271/sjweh.1313>

Winter, L., Geldmacher, J., Plücker-Boss, K., & Kahl, K. G. (2020). Integration of a Return-to-Work Module in Cognitive Behavioral Therapy in Patients With Major Depressive Disorder and Long-Term Sick Leave-A Feasibility Study. *Front Psychiatry*, *11*, 512. <https://doi.org/10.3389/fpsyt.2020.00512>
