# Supplementary material for: Work-focused cognitive behavioural therapy for common mental disorders: a systematic review and meta-analysis
Source: BMC Psychol. 2026 Sep 22;14:1374. doi: 10.1186/s40359-026-05648-2 (PMC13599331; doi:10.1186/s40359-026-05648-2)
Supplement: Supplementary file 2 [file 40359_2026_5648_MOESM2_ESM.docx]

**S1 Full search strategy**

1. Medline

| Interface: **Ovid MEDLINE(R) ALL** 1946 to December 05, 2025  Date of Search: 8 December 2025  Number of hits: 1,577  Comment: In Ovid, two or more words are automatically searched as phrases; i.e. no quotation marks are needed | Field labels   - exp/ = exploded MeSH term - / = non exploded MeSH term - .ti,ab,kf. = title, abstract and author keywords - adjx = within x words, regardless of order - * = truncation of word for alternate endings |
| --- | --- |
| **#** \| **Searches** \| **Results**; 1 \| exp Cognitive Behavioral Therapy/ \| 42695; 2 \| ((cogniti* or (acceptance adj3 commitment)) adj3 (intervention* or psychotherap* or therap* or treatment*)).ti,ab,kf. \| 59586; 3 \| (cbt or mindfulness).ti,ab,kf. \| 35687; 4 \| or/1-3 \| 89917; 5 \| Absenteeism/ \| 10252; 6 \| Employment/ \| 53314; 7 \| Employment, Supported/ \| 1456; 8 \| Occupational Health/ \| 38433; 9 \| Occupational Health Services/ \| 10844; 10 \| Occupational Medicine/ \| 23550; 11 \| Rehabilitation, Vocational/ \| 9964; 12 \| Return to Work/ \| 4368; 13 \| Sick Leave/ \| 7314; 14 \| Workplace/ \| 33678; 15 \| (absenteeism or medical leave* or (sick* adj2 absen*) or sick leave*).ti,ab,kf. \| 18482; 16 \| (occupational health or occupational medicine).ti,ab,kf. \| 27287; 17 \| (rehabilit* adj2 (employee* or employment or vocation* or work or worker*)).ti,ab,kf. \| 4678; 18 \| (work adj2 (absen* or return* or resuming or resumption)).ti,ab,kf. \| 20079; 19 \| ((vocation* or work-directed or work focused or workfocused or workplace or work-place or work related or workrelated) adj3 (integrat* or intervention* or program* or therap* or treatment*)).ti,ab,kf. \| 7637; 20 \| or/5-19 \| 203857; 21 \| 4 and 20 \| 1577 | |

2. APA PsycInfo

| Interface: Ebsco  Date of Search: 8 December 2025  Number of hits: 1,006  Comment: Search modes - Find all my search terms | Field labels   - DE = subject heading - TI = title - AB = abstract - KW = author keywords - Nx = within x words, regardless of order - * = truncation of word for alternate endings   Search mode and expanders:  Find all my search terms  Apply equivalent subjects |
| --- | --- |
| **S#** \| **Query (user-entered)** \| **Results (count)**; S1 \| DE "Cognitive Behavior Therapy" OR DE "Acceptance and Commitment Therapy" OR DE "Cognitive Processing Therapy" OR DE "Prolonged Exposure Therapy" \| 31523; S2 \| DE "Mindfulness" \| 15659; S3 \| TI ( (cogniti* OR (acceptance N2 commitment)) N2 (intervention* OR psychotherap* OR therap* OR treatment*)) ) OR AB ( (cogniti* OR (acceptance N2 commitment)) N2 (intervention* OR psychotherap* OR therap* OR treatment*)) ) OR KW ( (cogniti* OR (acceptance N2 commitment)) N2 (intervention* OR psychotherap* OR therap* OR treatment*)) ) \| 67696; S4 \| TI ( (cbt OR mindfulness) ) OR AB ( (cbt OR mindfulness) ) OR KW ( (cbt OR mindfulness) ) \| 43235; S5 \| S1 OR S2 OR S3 OR S4 \| 92697; S6 \| DE "Employee Absenteeism" \| 2567; S7 \| DE "Employment Status" OR DE "Employability" OR DE "Employment History" OR DE "Job Loss" OR DE "Reemployment" OR DE "Retirement" OR DE "Self-Employment" OR DE "Unemployment" \| 36806; S8 \| DE "Vocational Rehabilitation" OR DE "Supported Employment" OR DE "Vocational Evaluation" OR DE "Work Adjustment Training" \| 8493; S9 \| DE "Occupational Health" OR DE "Work Related Illnesses" \| 9525; S10 \| DE "Occupational Health Psychology" \| 264; S11 \| DE "Employee Leave Benefits" \| 1822; S12 \| TI "Occupational Medicine" OR AB "Occupational Medicine" OR KW "Occupational Medicine" OR TI "Occupational health" OR AB "Occupational health" OR KW "Occupational health" \| 4991; S13 \| TI ( absenteeism OR "medical leave*" OR (sick* N1 absen*) OR "sick leave*") ) OR AB ( absenteeism OR "medical leave*" OR (sick* N1 absen*) OR "sick leave*") ) OR KW ( absenteeism OR "medical leave*" OR (sick* N1 absen*) OR "sick leave*") ) \| 8482; S14 \| TI ( (rehabilit* N1 (employee* OR employment OR vocation* OR work OR worker*)) ) OR AB ( (rehabilit* N1 (employee* OR employment OR vocation* OR work OR worker*)) ) OR KW ( (rehabilit* N1 (employee* OR employment OR vocation* OR work OR worker*)) ) \| 6192; S15 \| TI ( (work N1 (absen* OR return* OR resuming OR resumption)) ) OR AB ( (work N1 (absen* OR return* OR resuming OR resumption)) ) OR KW ( (work N1 (absen* OR return* OR resuming OR resumption)) ) \| 5556; S16 \| ( TI ((vocation* OR "work-directed" OR "work focused" OR workfocused OR workplace OR "work-place" OR "work related" OR workrelated) N2 (integrat* OR intervention* OR program* OR therap* OR treatment*)) ) OR ( AB ((vocation* OR "work-directed" OR "work focused" OR workfocused OR workplace OR "work-place" OR "work related" OR workrelated) N2 (integrat* OR intervention* OR program* OR therap* OR treatment*)) ) OR ( KW ((vocation* OR "work-directed" OR "work focused" OR workfocused OR workplace OR "work-place" OR "work related" OR workrelated) N2 (integrat* OR intervention* OR program* OR therap* OR treatment*)) ) \| 6974; S17 \| S6 OR S7 OR S8 OR S9 OR S10 OR S11 OR S12 OR S13 OR S14 OR S15 OR S16 \| 69582; S18 \| S5 AND S17 \| 1006 | |

3. Web of Science Core Collection

| Interface: Clarivate Analytics  Date of Search: 8 December 2025  Number of hits: 1,454 | Field labels   - TS/Topic = title, abstract, author keywords and Keywords Plus - NEAR/x = within x words, regardless of order - * = truncation of word for alternate endings   Note: the *Exact search*-function was used for all the searches |
| --- | --- |
| # \| Search Query \| Results; 1 \| TS=((cogniti* OR (acceptance NEAR/2 commitment)) NEAR/2 (intervention* OR psychotherap* OR therap* OR treatment*) ) \| 94861; 2 \| TS=(cbt OR mindfulness) \| 60551; 3 \| #1 OR #2 \| 131468; 4 \| TS= "occupational health" \| 29159; 5 \| TS="occupational medicine" \| 4109; 6 \| TS=(absenteeism OR "medical leave*" OR (sick* NEAR/1 absen*) OR "sick leave*") \| 24679; 7 \| TS=(rehabilit* NEAR/1 (employee* OR employment OR vocation* OR work OR worker*) ) \| 6913; 8 \| TS=(work NEAR/1 (absen* OR return* OR resuming OR resumption) ) \| 20808; 9 \| TS=((vocation* OR "work-directed" OR "work focused" OR workfocused OR workplace OR "work-place" OR "work related" OR workrelated) NEAR/2 (integrat* OR intervention* OR program* OR therap* OR treatment*) ) \| 10860; 10 \| #4 OR #5 OR #6 OR #7 OR #8 OR #9 \| 84841; 11 \| #3 AND #10 \| 1454 | |
