# Supplementary material for: Work-focused cognitive behavioural therapy for common mental disorders: a systematic review and meta-analysis
Source: BMC Psychol. 2026 Sep 22;14:1374. doi: 10.1186/s40359-026-05648-2 (PMC13599331; doi:10.1186/s40359-026-05648-2)
Supplement: Supplementary file 4 [file 40359_2026_5648_MOESM4_ESM.docx]

**S3 Additional Information**

**Additional Information for W-CBT meta-analysis of RTW intervention studies (Finnes et al., 2026)**

Figures 1, 2, and 3 show rated items on the y-axis and studies on the x-axis, both ordered by total score. Items least frequently reported or applied appear at the bottom of the y-axis, and studies with higher total scores are further to the right on the x-axis.

Figure 1. Treatment content analysis. Items (y axis) and studies (x axis) are sorted on total scores.

Figure 2. Work focused content analysis. Items (y axis) and studies (x axis) are sorted on total scores.

Figure 3. Research methodology scores for all included studies. Items (y axis) and studies (x axis) are sorted on total scores.

Figure 4. Forest plot for attrition rates at post.

Table 1. Risk of bias overall ratings for all included studies.

| Bias rating tool | Study | Rating |
| --- | --- | --- |
|  | Blonk, 2006 | Some concerns |
|  | Brouwers, 2006 | Some concerns |
|  | Dalgaard, 2017 | Some concerns |
|  | deWeerd, 2015 | Some concerns |
|  | Finnes, 2019 | Low |
|  | Geraedts, 2014 | Low |
|  | Himle, 2014 | Some concerns |
|  | Hoff, 2021 | Low |
| RoB2 | Kröger, 2015 | High |
|  | Lerner, 2015 | Low |
|  | Noordik, 2013 | High |
|  | Reme, 2015 | Low |
|  | Salomonsson, 2017 | Some concerns |
|  | van der Klink, 2003 | Some concerns |
|  | Willert, 2009 | Some concerns |
|  | Gjengedal, 2025 | Low |
|  | Corbiere, 2021 | Serious |
|  | Gjengedal, 2020 | Serious |
|  | Ito, 2019 | Critical |
| ROBINS-I | Lagerveld, 2012 | Serious |
|  | Winter, 2020 | Serious |
|  | Bond, 2025 | Serious |
|  | Monsen, 2025 | Moderate |

Table 2. RoB2 ratings.

| **Study** | **Randomization** | **Missing data** | **Measurement of outcome** | **Selection of the results** | |
| --- | --- | --- | --- | --- | --- |
| Blonk, 2006 | Some concerns | Low | Low | Some concerns | |
| Brouwers, 2006 | Low | Low | Low | Some concerns | |
| Dalgaard, 2017 | Some concerns | Low | Low | Some concerns | |
| deWeerd, 2015 | Low | Some concerns |  |  | |
| Finnes, 2019 | Low | Low | Low | Low | |
| Geraedts, 2014 | Low | Low | Low | Low | |
| Himle, 2014 | Low | Low | Low | Some concerns | |
| Hoff, 2021 | Low | Low | Low | Low | |
| Kröger, 2015 | High | Low | Low | Some concerns | |
| Lerner, 2015 | Low | Low | Low | Low | |
| Noordik, 2013 | High | Low | Low | High | |
| Reme, 2015 | Low | Low | Low | Low | |
| Salomonsson, 2017 | Low | Low | Low | Low | |
| van der Klink, 2003 | Some concerns | Low |  | Some concerns | |
| Willert, 2009 | Low | Low | Low | Some concerns | |
| Gjengedal, 2025 |  |  |  |  |  |

Table 3. ROBINS-I ratings.

| **Study** | **Confoun-ding** | **Selection bias** | **Classification of interventions** | **Deviations from intended interventions** | **Missing data** | **Measure-ments of outcomes** | **Selection of the results** |
| --- | --- | --- | --- | --- | --- | --- | --- |
| Bond, 2025 | Critical | Low risk | Low | Low | Serious | Serious | Moderate |
| Corbiere, 2021 | Moderate | Low risk | Low | Low | Low | Serious | Moderate |
| Gjengedal, 2020 | Serious | Low risk | Moderate | Low | Serious | Serious | Moderate |
| Ito, 2019 | Critical | Low risk | Low | Low | Moderate | Serious | Moderate |
| Lagerveld, 2012 | Moderate | Low risk | Low | Low | Serious | Serious | Moderate |
| Monsen 2025 | Critical | Low risk | Low | Low | Moderate | Moderate | Moderate |
| Winter, 2020 | Critical | Low risk | Low | Low | Low | Serious | Moderate |

Figure 5. Z-curve evaluating publication bias using random effects and robust Bayesian meta-analysis (RoBMA).
